# Born in Bradford’s Better Start: an experimental birth cohort study to evaluate the impact of early life interventions

**DOI:** 10.1186/s12889-016-3318-0

**Published:** 2016-08-04

**Authors:** Josie Dickerson, Philippa K. Bird, Rosemary R. C. McEachan, Kate E. Pickett, Dagmar Waiblinger, Eleonora Uphoff, Dan Mason, Maria Bryant, Tracey Bywater, Claudine Bowyer-Crane, Pinki Sahota, Neil Small, Michaela Howell, Gill Thornton, Melanie Astin, Debbie A. Lawlor, John Wright

**Affiliations:** 1 Born in Bradford, Bradford Institute for Health Research, Bradford Teaching Hospitals NHS Foundation Trust, Bradford, BD9 6RJ UK; 2 Department of Health Sciences, The University of York, York, UK; 3 Leeds Institute of Clinical Trials Research, University of Leeds, Leeds, UK; 4 Psychology in Education Research Centre, Department of Education, The University of York, York, UK; 5 School of Clinical and Applied Sciences, Leeds Beckett University, Leeds, UK; 6 Faculty of Health Studies, University of Bradford, Bradford, UK; 7 Better Start Bradford, Bradford Trident, Bradford, UK; 8 BD4 Family, BD4 Community Trust, Bradford, UK; 9 MRC Integrative Epidemiology Unit at the University of Bristol and School of Social and Community Medicine, University of Bristol, Bristol, UK

**Keywords:** Birth cohort, Trials within cohort, Quasi-experimental, Inequalities, Early years interventions, Child health, Child development, Social, Emotional, Communication, Language, Obesity, Nutrition

## Abstract

**Background:**

Early interventions are recognised as key to improving life chances for children and reducing inequalities in health and well-being, however there is a paucity of high quality research into the effectiveness of interventions to address childhood health and development outcomes. Planning and implementing standalone RCTs for multiple, individual interventions would be slow, cumbersome and expensive. This paper describes the protocol for an innovative experimental birth cohort: Born in Bradford’s Better Start (BiBBS) that will simultaneously evaluate the impact of multiple early life interventions using efficient study designs. Better Start Bradford (BSB) has been allocated £49 million from the Big Lottery Fund to implement 22 interventions to improve outcomes for children aged 0–3 in three key areas: social and emotional development; communication and language development; and nutrition and obesity. The interventions will be implemented in three deprived and ethnically diverse inner city areas of Bradford.

**Method:**

The BiBBS study aims to recruit 5000 babies, their mothers and their mothers’ partners over 5 years from January 2016-December 2020. Demographic and socioeconomic information, physical and mental health, lifestyle factors and biological samples will be collected during pregnancy. Parents and children will be linked to their routine health and local authority (including education) data throughout the children’s lives. Their participation in BSB interventions will also be tracked. BiBBS will test interventions using the Trials within Cohorts (TwiCs) approach and other quasi-experimental designs where TwiCs are neither feasible nor ethical, to evaluate these early life interventions. The effects of single interventions, and the cumulative effects of stacked (multiple) interventions on health and social outcomes during the critical early years will be measured.

**Discussion:**

The focus of the BiBBS cohort is on intervention impact rather than observation. As far as we are aware BiBBS is the world’s first such experimental birth cohort study. While some risk factors for adverse health and social outcomes are increasingly well described, the solutions to tackling them remain elusive. The novel design of BiBBS can contribute much needed evidence to inform policy makers and practitioners about effective approaches to improve health and well-being for future generations.

**Electronic supplementary material:**

The online version of this article (doi:10.1186/s12889-016-3318-0) contains supplementary material, which is available to authorized users.

## Background

This paper describes the protocol for an innovative experimental birth cohort: Born in Bradford’s Better Start (BiBBS) to evaluate the effects of multiple early life interventions on social and emotional development, communication and language development, and nutrition and obesity. Early interventions are recognised as key to improving life chances for children and reducing inequalities in health and well-being [1–3]. Whilst there is evidence about the factors associated with risk and resilience in early childhood health and development [4, 5], there is a paucity of high quality research into the effectiveness of interventions to address them and improve outcomes [6]. Planning and implementing standalone RCTs for multiple, individual interventions would be slow, cumbersome and expensive. In the meantime our next generation of children are growing up facing the same inequalities and poor health. Policy makers and practitioners need evidence now if they are to enable healthier futures.

The BiBBS cohort will evaluate the impact of multiple interventions on the critical early years of life. Birth cohorts are traditionally observational epidemiological studies used to elucidate factors associated with health outcomes. Our focus in BiBBS is on intervention rather than observation, and so we refer to this novel methodological approach as an “experimental birth cohort study”. We are not aware of any other birth cohorts applying this design, making BiBBS the world’s first experimental birth cohort study.

The early years of life are critical in determining physical, emotional and cognitive development [4]. What happens in early childhood can have a lifelong effect on health and well-being—from physical health, including obesity and heart disease, and mental health through to educational attainment and economic status [5]. The UK’s latest independent review of health inequalities, Fair Society, Healthy Lives, based on more than three decades of research on the social determinants of health and health inequalities, recommended “giving every child the best start in life” as the highest priority [5].

There is a need for robust evaluations of early years interventions to improve health and well-being. Whilst randomised controlled trials (RCTs) are considered a ‘gold standard’ of intervention evaluation [7], they generally focus on a single intervention, are both time consuming and expensive to complete [8], and may not be ethical or feasible for complex early years interventions [9, 10]. There has been a growing interest in natural experiments, using cohort and other observational datasets, as a more feasible and ethical method to evaluate complex interventions [10, 11]. Natural experiments frequently use routinely collected data to assess outcomes thereby allowing more efficient evaluations that are relevant to local health and social care [12–14].

The BiBBS experimental birth cohort will use a range of designs including the randomised controlled Trials within Cohorts (TwiCs) approach [15], and other methods including quasi-experimental designs where TwiCs are neither feasible nor ethical to evaluate multiple early years interventions. The BiBBS cohort also provides the unique opportunity to investigate the combined effects of stacked (multiple, layered) interventions. The BiBBS cohort will link routinely collected health and educational data of the participants, allowing an efficient collection of outcome data on a scale that would not be possible using other research methods. The use of such data also ensures that the key outcomes are relevant to policy and practice.

The early years interventions that will be evaluated as a part of the BiBBS cohort are delivered within the context of a natural social and public health experiment—the Better Start Bradford (BSB) programme. In 2015 the UK’s Big Lottery funded the ‘A Better Start’ initiative to improve the life chances of over 60,000 babies and young children living in some of the poorest parts of England. A total of £215 million has been allocated to the initiative in five areas of England (Blackpool, Bradford, Nottingham, Lambeth, Southend). Each area will undertake a variety of programmes to improve outcomes for children in three key areas: social and emotional development; communication and language development; and nutrition and obesity.

BSB has been allocated just under £49 million to implement 22 interventions in three inner city areas of Bradford over a 10 year period from 2015 to 2025. The BSB programme aims to implement evidence based interventions, however the lack of high quality research [6] for the effectiveness of early life interventions made selection of appropriate programmes challenging. Following a comprehensive review [6] a range of ‘evidence based’ (defined as tested and proven effective using robust study designs (systematic reviews or RCTs)) and ‘science based’ interventions (defined as developed using the best available evidence, but not tested or proven effective using robust methods of evaluation) were selected for implementation in BSB. Of the 22 interventions, two are backed by RCT evidence and 20 are science based.

The interventions include additional support for teenage mothers, reduced midwifery caseloads, a befriender scheme for all mothers affected by or at risk of postnatal depression, language development programmes, storytelling groups, outdoor play and exercise activities, breastfeeding support and healthy lifestyle and parenting programmes. New parents will be introduced to local Children’s Centres and a targeted service will work with them to increase their understanding of infant development. Simultaneously, community initiatives will improve the local environment for children and families in the area and systems changes will be implemented to radically alter child health and early years’ services. Each of the interventions will go through a service design process to ensure that the intervention is responsive to the needs of the local community. A summary of the interventions can be seen in Table 1.

**Table 1:** Interventions to be delivered as a part of Better Start Bradford

Intervention	Description	Estimated no. recipients (over 5 years)^c^	Main outcome domain^d^
Antenatal Support			
Personalised Midwifery	Continuous midwife care	1250	Social & Emotional Development /Obesity & Nutrition
Baby Buddy/ Best Beginnings	Phone app	2500	
Family Links Antenatal	Universal antenatal parenting skills programme	2500	
Doula	Pregnancy support for vulnerable women	300	
HAPPY	Healthy eating & parenting course for overweight mums	1050	
Pregnancy & breast feeding	Peer support for breastfeeding	2250	Obesity & Nutrition
Antenatal & Postnatal Support			
Family Nurse Partnership^a^	Nurse-led support for teenage pregnant women	500	Social & Emotional Development
Baby Steps Antenatal + Postnatal	Antenatal support for women at risk of poor emotional well-being	500	
Family Action Perinatal Peer Support	Peer support for mothers with mild/moderate mental health issues	450	
ESOL+	English language course for parents	950	Communication & Language Development
Postnatal Support			
Infant Mental Health	Professional support for women with poor attachment	200–500	Social & Emotional Development
Northamptonshire Baby Room	Infant brain development course	580	
Incredible Years Parenting ^b^	Parenting programme	1400	
Home-Start	Volunteer support for vulnerable women	225	
Family Links Nurturing	Parenting skills programme for vulnerable families	1050	
Early Years Support			
HENRY	Universal lifestyle programme for parents with young children	1050	Social & Emotional Development /Obesity & Nutrition
Community Nutrition Skills	Cook and eat sessions	1000	Obesity & Nutrition
PiP	Pre-schoolers physical activity in the playground	1500	
Forest Schools	Outdoor play for young children & parents	1500	
Bookstart and Imagination Library	Book gifting service age 0–5	7000	Communication & Language Development
I CAN	Children aged 2 at risk of language delay	675	
Talking Together	Sessions for parents with children aged 2 with language delay	2075	

^a^Evidence based from US study, recently proved non-effective in UK setting

^b^Evidence based intervention (all other interventions are science based)

^c^Estimated number of recipients are taken from the original BSB bid to the BLF. Actual numbers will be finalised within the service design process, based on consideration of local need and service capacity

^d^The specific outcomes for each intervention will be finalised in the service design process

A key component of the BSB programme is the BSB Innovation Hub, a collaboration of Born in Bradford and BSB, that will provide a centre for evaluation of the 22 interventions. Born in Bradford (BiB) is an ongoing birth cohort study which recruited over 13,500 babies born across the city between 2007 to 2011 [14, 16]. BSB is a community partnership led by Bradford Trident, a community-led social enterprise providing support for the local community. The roles of BSB and the Innovation Hub are distinct, with the partners of BSB independently selecting, co-designing, commissioning and implementing the interventions, and the Innovation Hub independently evaluating these selected interventions. The aim of the Innovation Hub is to further our understanding of whether the chosen interventions are effective in the BSB context, and how the interventions work in combination within the framework of the new BiBBS birth cohort study. To achieve this the Innovation Hub will adopt a flexible and responsive approach using a number of different methods that will be tailored to each intervention as it is designed and implemented in the local community.

### Study aims and objectives

The aim of BiBBS is to evaluate the effects of multiple early life interventions on social and emotional development, communication and language development, and nutrition and obesity. This will be achieved by following families from pregnancy into childhood using linkage to routinely collected data. Our goal is to contribute robust evidence to policy makers, practitioners and local communities that will help inform health policy and planning, locally, nationally and internationally.

The cohort will also provide a strong foundation for future research into children’s health and development, including the roles of social and environmental factors, such as ethnicity, poverty and neighbourhood deprivation, and behavioural factors. It will include biobank resources to allow for the investigation of physiological, hormonal, genetic and epigenetic pathways.

This protocol outlines the study methods and design considerations for the initial phase of this experimental cohort study of mothers, their babies and their partners living in the BSB areas of inner city Bradford.

## Methods

The protocol for recruitment and collection of baseline and routine outcome data and biological samples for the cohort has been approved by Bradford Leeds NHS Research Ethics Committee (15/YH/0455). Research governance approval has been provided from Bradford Teaching Hospitals NHS Foundation Trust.

**Fig. 1:**
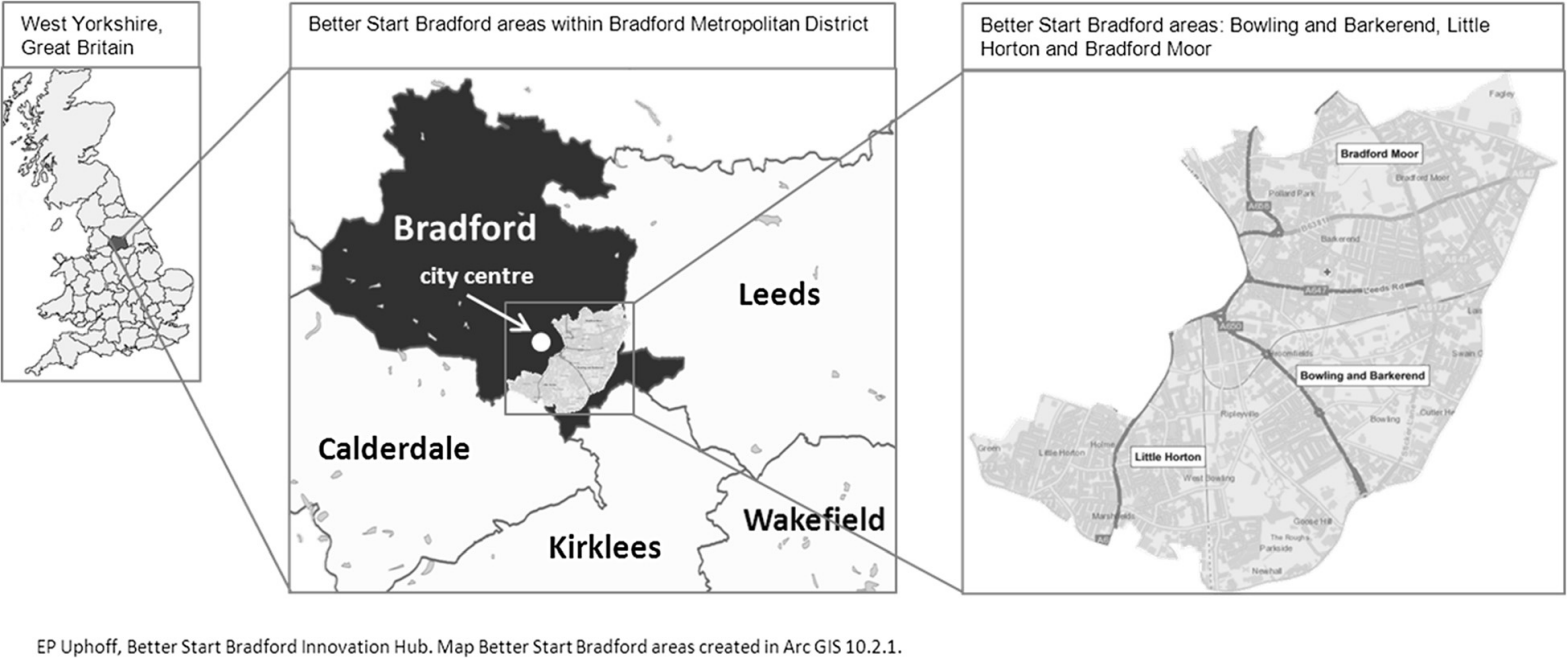
Location map of Bradford and BSB areas

### Setting

Bradford district, located in West Yorkshire in the North of England, is the 6^th^ largest district in England and is the 19^th^ most deprived local authority of 326 in England [17]. The Better Start Bradford area comprises three inner city areas of Bowling and Barkerend, Bradford Moor and Little Horton (see Fig. 1). The majority of the BSB area falls into the most deprived 10 % of areas in England [17]. The demographic characteristics of the BSB areas compared with Bradford and England are presented in Table 2. In summary, the three areas of BSB make up 12.3 % of the population of Bradford and are among the most deprived in the Bradford district and in England. BSB areas have a higher birth rate than Bradford district or England. The BSB areas are very ethnically diverse, with residents of Pakistani heritage forming the largest ethnic group (48.6 %) and a White British population of 24.8 %. An increasing number of families arriving from a range of central and eastern European countries, especially Poland, Slovakia and the Czech Republic, add to the diversity of the areas. Mortality and morbidity rates in these areas are higher than in Bradford district and England, and include a high infant mortality rate. There are higher rates of obesity and extremely poor oral health compared with Bradford district and England.

#### Community engagement

A major strength of the original BiB cohort is the compelling track record of local community involvement. The Bradford community and local parents are at the heart of the BiB research programme [18]. The BiBBS cohort is committed to continuing this ethos.

BiBBS has established a Community Representatives Advisory Group (CRAG) made up of community representatives from the BSB areas including BSB engagement workers, local parents, leaders of local groups, projects and charities and local councillors. This group has been involved at every stage of development from study design including the development of the baseline questionnaires, information sheets and consent forms and methods for engaging with and recruiting parents. The CRAG will continue to work in partnership with BiBBS throughout recruitment by helping to engage with the local community and provide feedback on successes and challenges. The CRAG will also play a key role in the interpretation and dissemination of findings.

### Eligibility

#### Inclusion criteria

##### Pregnant Women

All pregnant women living in BSB areas (defined by full postcode) who are registered to give birth at Bradford Teaching Hospitals NHS Foundation Trust (BTHFT) will be eligible for recruitment. The BTHFT is the only maternity unit covering this area.

##### Babies

All babies born to women who have consented to participate in the cohort study will be included in the cohort.

##### Partners

The partners of the women who have consented to take part will also be invited to participate. In the majority of cases this will be the baby’s father; however, the primary interest for the birth cohort is with the women’s co-habiting partner rather than the biological father.

Women and partners can take part in the study for each pregnancy that occurs whilst they live in the BSB area, during the recruitment timeframe.

#### Exclusion criteria

##### Pregnant Women

Women will be excluded if they plan to move away from Bradford before the birth.

##### Partner

If a participating woman does not want the research team to approach their partner then they will not be recruited.

### Sample size

The BiBBS study aims to recruit 5000 babies over 5 years from January 2016 to December 2021. All women booked for delivery at BTHFT are offered an oral glucose tolerance test (GTT) at 26 to 28 weeks gestation. As 75 % of women attend for the GTT [14] the majority of women and their partners will be recruited in these clinics. For those who do not attend, recruitment will take place in community settings (e.g. at midwife appointments) and at other hospital appointments (e.g. diabetes clinics). Although every effort will be made to find and approach all eligible women, we predict reaching approximately 85 % of the eligible population through these methods. We assume, based on analysis of BiB data [14] and BTHFT data, that:

1. 1450 babies are born in the BSB area per year,
2. 85 % of pregnant women will be reached and invited to take part (75 % at GTT, 10 % in other settings),
3. 80 % of these women will agree to take part.
4. In BiB, the ratio of babies to mothers was 1.11, due to multiple births and multiple pregnancies [14].

As such, we expect to recruit 4930 babies and 4440 mothers over a 5 year period.

Uptake for the partners is likely to be lower and we expect, based on achieved recruitment in BiB, to recruit at least 25 % of partners (*n* = 1125).

### Identification and information provision

#### Pregnant women

**Fig. 2:**
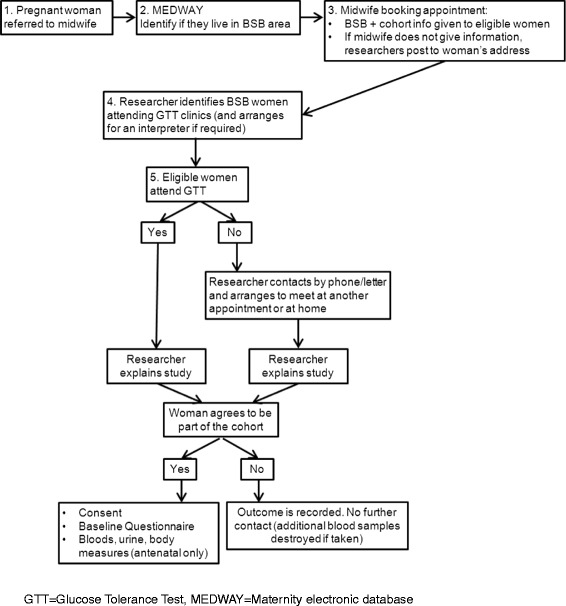
The recruitment process

A system has been developed to flag all women living in the BSB areas, determined by full postcode, on the electronic maternity system. When women attend their first appointment (around 10–12 weeks gestation), the BSB flag will prompt midwives to provide women with information on BSB and the BiBBS cohort study and obtain verbal assent to data sharing with the BiBBS research team. A flow-chart showing the recruitment process for women and babies is provided in Fig. 2.

Recruitment will be completed by trained researchers who are not involved in the women’s clinical care. Researchers will identify eligible women who have provided assent to data sharing, and will approach women during the GTT clinic to invite them to participate in the cohort. Women who do not attend or who are not approached during the GTT will be approached at another appointment (e.g. community midwife appointments). If this is not possible, the research team will phone to invite them to meet a researcher at a time and place convenient to the woman, for example at an appointment in a local clinic or in their own home. For late presenting women the approach will be when they are an inpatient on the maternity wards.

An anonymised screening log will be maintained to record the woman’s age, ethnicity, language spoken, expected due date, parity, attendance at GTT, and BiBBS status (whether they have been approached, whether they have consented and reason for refusal). This will enable the recruitment of women from all ethnic backgrounds, languages, and age groups to be monitored.

#### Partners

Partners will be invited to take part using a variety of methods. Where partners attend the booking appointment, a partner information sheet will be given to them. Researchers will try to recruit partners face to face, either at the GTT clinic, at other appointments, or on the maternity unit following the birth of the child. If researchers are not able to contact partners face to face, they will ask participating women to take a recruitment pack home for their partners to complete.

### Language needs

The information sheets for pregnant women and for partners of pregnant women have been carefully developed for the population of BSB areas, with advice and feedback from members of the BSB community. They have a Flesch reading score of 64 (suitable for ages 12 upwards) and are appropriate for people with basic English as well as those under the age of 16 [19].

A large proportion (29 %) of the population in the BSB areas do not speak or read English. Information sheets, consent forms and baseline questionnaires will be translated/transliterated into the most common languages spoken by women requiring language support in the BSB areas: Urdu, Punjabi/Mirpuri, Slovakian, Polish, Sylheti. The translation process can be seen in detail in Additional file 1. Audio recorded spoken versions of the information sheet and consent forms will be produced for languages without a written form, or for those who are unable to read the language (Urdu, Mirpuri/Punjabi, Sylheti).

Interpreters will be used to facilitate inclusion of women who speak languages that are less common in the BSB area. Researchers will identify women’s language needs and will make use of the interpreters who attend the clinic / appointment with the woman to explain the study, take consent and translate a shortened version of the baseline questionnaire.

### Consent

Informed consent will be obtained from expectant women (including consent for the baby/babies that the mother is expecting) and their partners.

Women and/or partners aged under 16 will be consented if deemed Gillick competent (having the intelligence and maturity to understand the research and the ability to understand the implications of that decision) [20]. Where the woman or partner is not able to consent themselves, their parent/guardian will be involved in the recruitment process and will be asked to provide consent for their child/ward’s participation. Consent will not be taken from any woman or partner aged 16 or over who is deemed not competent [21].

#### Withdrawals, deaths and changes in primary carers

Participants can contact the BiBBS office to request withdrawal from the cohort study at any time. BiBBS office staff will carefully collect information in order to check the type of withdrawal category (e.g. of future contact / deletion of all existing data) with the participant.

On receipt of notification of a miscarriage, stillbirth or child death, the woman and child will automatically be withdrawn from BiBBS. Samples and data collected up to that point will be retained.

In the case of a maternal death or adoption, after an appropriate time (usually 6 months), an attempt will be made to identify and consent the new main carer/guardian. If a child moves into foster care or between foster carers, attempts to identify a new long-term carer/guardian will be made every 6 months. Once a new main carer/guardian is identified the research team will attempt to consent them.

### Data collection

Following consent, the women will be invited to complete a baseline questionnaire and provide a blood sample, urine sample and have anthropometric measurements and carotenoid levels (a biomarker of antioxidant levels, indicating fruit and vegetable consumption) taken. Partners will complete the baseline questionnaire and provide an optional saliva sample and have anthropometric measurements taken.

### Baseline questionnaire

A baseline questionnaire has been developed to provide information to support the evaluation of the BSB interventions. Key components include:

- Household information
- Socioeconomic status
- Ethnic, cultural and religious background
- Social, demographic and family information
- Neighbourhood
- Physical and mental health
- Language and Communication skills including home literacy environment
- Behaviour/lifestyle factors including eating habits.

Where available and appropriate, validated questionnaires have been used. Additional sections have been developed based on the BiB cohort questionnaire [16] and through expert opinion of the authors and the local BSB community. The full questionnaire can be seen in Additional file 1. A shortened version of the questionnaire (Additional file 1) will be used where an interpreter is required. The partners questionnaire is a shortened version of the baseline questionnaire used for women and can be seen in Additional file 1.

The baseline questionnaire for women is designed to be partly self-completed and partly administered by a researcher using a tablet device. Researchers will sit with the women while they complete the self-completion questions and will be able to answer any queries. At the end of the questionnaire automated flags will indicate any safeguarding or mental health concerns and the researchers will be prompted to follow standard protocols.

The baseline Questionnaire for partners will be self-completed electronically or via a postal questionnaire.

#### Samples and measurements

Women and partners will consent to the collection of different biological samples and measurements. These are summarised in Table 3 and further details of collection, processing and storage of biological samples can be seen in Additional file 1.

**Table 2:** Demographic Information of the Better Start Bradford area compared to Bradford and England

	Better Start Bradford	Bradford district	England
Population (all ages) [34]	65102	528155	54316618
Births per year (rate per 1000 population) [35]	1335 (20)	8100 (15)	661501 (12)
Children 0-3 years (% of the population) [34]	5467 (8.4 %)	32711 (6.2 %)	2748017 (5.1 %)
Ethnicity [36]			
White British	24.8 %	63.9 %	79.8 %
Asian/Asian British: Pakistani	48.6 %	20.4 %	2.1 %
Asian/Asian British: Bangladeshi	5.2 %	1.9 %	0.8 %
Asian/Asian British: Indian	3.8 %	2.6 %	2.6 %
White: Other White	3.7 %	3.0 %	4.6 %
Black	3.2 %	1.8 %	3.4 %
Other ethnicities	10.7 %	6.4 %	6.7 %
Infant mortality rate (per 1000 live births) [37]	^a^ 9.4	7.0	3.6
Decayed, missing, filled teeth (Mean d3mft) age 5 [38]	^b^ 3.6	1.98	0.94
Obesity [39, 40]			
Reception (age 4–5)	11.0 %	8.6 %	9.1 %
Year 6 (age 10–11)	25.7 %	21.5 %	19.1 %

^a^Source: https://www.whatdotheyknow.com/request/infant_mortality_rates_in_bradfo; Provided by a Bradford City Council Public Health Information Analyst in response to Freedom of Information request; data from Better Start Wards are combined to provide IMR per 1000 live births from 2004 to 2012

^b^Ward level data provided by Bradford City Council Public Health

#### Routine data collection

**Fig. 3:**
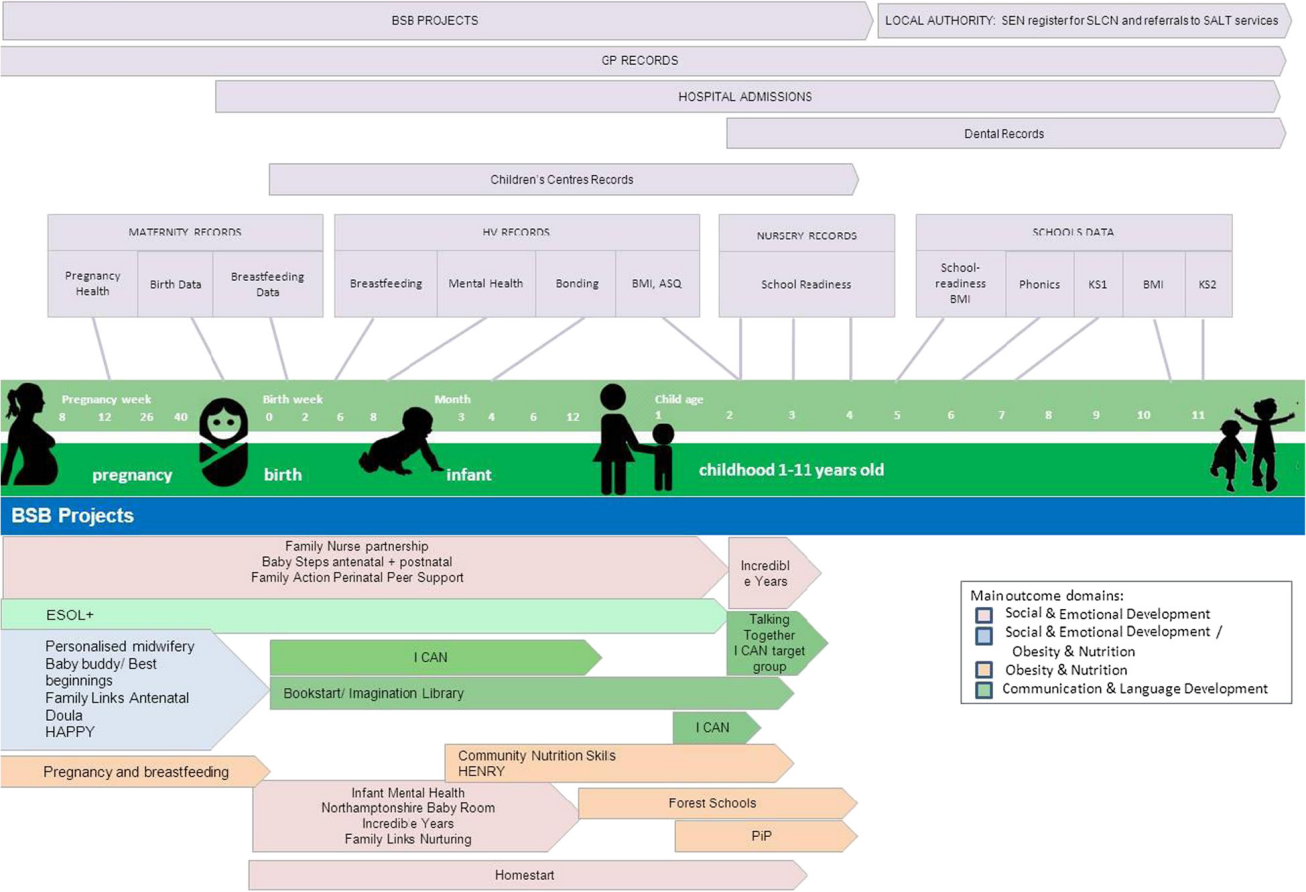
Routine outcome measures

A summary of the planned routine data linkage from health, local authority and BSB interventions can be seen in Fig. 3. Data will be linked by NHS number for healthcare records and BSB intervention data, by Unique Pupil Number (UPN) for education records, and by a unique E-Start number for Children’s Centres. Data sharing protocols will be agreed with all organisations.

#### Unique study identifier

Each participant will be allocated a unique identification number which will be used to identify their data throughout the study. Women, their babies and partners will be linked together using a pregnancy identification number. NHS numbers will also be collected for each participant to facilitate data linkage. Families’ attendance at BSB interventions will be tracked using their NHS and UPN numbers. This will allow the cohort to follow families’ journeys through the different interventions. We will use a secure database hosted at the BTHFT to store all of the cohort data on individual children and their parents.

### Retention

As planned follow-up data will all be routinely collected, retention rates will depend on the availability and linkage with routine data. In the BiB study, 86 % of children in Bradford have been linked to schools and over 99 % of mothers and children have been linked with GP records, and the same success rate is expected in BiBBS [18].

To retain families and to keep them engaged, an annual newsletter and birthday card will be sent to each family. This was a successful element of the ongoing BiB cohort, and will be repeated for the new BiBBS cohort. The newsletter will gather the highlights from a BiBBS website over the year, focussing on celebrating key achievements, and findings from the research. Data linkage to GP records ensures that home addresses and the survival status of all participants are regularly updated on the database allowing newsletters and birthday cards to be sent to the most recent address and to be not sent where there has been a recent death.

### Data quality and confidentiality

#### Baseline questionnaire and optional samples/measurements

Researchers will receive formal training from experts in the field of data collection including methods and techniques used in collecting data, practical demonstrations and role play. Data collection will be observed by an independent observer on a regular basis for quality assurance. Researchers will have up to date Good Clinical Practice (GCP) training and will be trained in administering the questionnaire. They will be provided with detailed instructions and background information. Researchers will be supported by the BiB research midwife who will be available for advice and guidance. They will also be trained in BTHFT wide policies on lone working and safeguarding of adults and children.

#### Routine data collection and measurements

Researchers, paediatricians, hospital midwives and health visiting teams in the community will receive regular training on the measures that are included in the cohort. This includes the standard anthropometric techniques needed to assess adult and infant size, assessments of mother-child relationship, maternal depression and anxiety, breastfeeding, routine biological samples and other measures. The training will focus on the importance of reliable data collection and protocols for measurements or assessments in order to reduce error.

All information collected during the course of the cohort will be kept strictly confidential. Information will be held securely on paper and electronically. BiBBS will comply with all aspects of the 1998 Data Protection Act [22]. Wherever possible, consent and baseline questionnaires will be completed electronically using tablet devices. The data collected will be saved onto the BTHFT secure computer server and will not be stored on the local device. Data will only be accessible by authorised members of the BiB team. Data will only be stored with personal identifiers if absolutely necessary. Where electronic data collection is not possible, paper consents and questionnaires will be used and then entered onto the database held within the BTHFT secure computer server. Paper files containing personal identifiers will be stored in locked cabinets within BIHR, separate from all other data.

### Data management

Primary data captured by electronic forms will be synchronised with the cohort database to verify identifiers and validate newly captured data against existing data. Automatic interactive validation will be built into individual data item inputs in electronic forms and questionnaires, such as logical traps, format validations, range limiters and, automated questionnaire flow with mandatory values. Electronic data capture clients for primary data and biosample tracking data will synchronise with the cohort database, from which unattended transform and report functions will provide regular datasets for researchers to analyse to ensure data quality standards are being met. Record matches for routine data linkage will be validated on the basis of unique identifiers (e.g. NHS number) plus multiple non-unique identifiers (e.g. surname, date of birth) where possible. Where unique identifiers are not available, iterative deterministic matching on the basis of multiple sets of non-unique identifiers will be used. The central database, hosted by BTHFT, will store data obtained from all sources listed under *Data Collection*. Data from each source will be linked at the BiBBS person level and will be structured and maintained by BiBBS data managers as a long term strategic store to service cohort data capture, analysis and other research activities as necessary. The entire database schema and data will be backed up nightly. Further details of the data management process can be seen in Additional file 1.

### Methods for analysis

The BiBBS experimental cohort design will enable evaluation of the BSB interventions using both experimental and quasi-experimental methods, in addition to traditional epidemiological approaches to the analysis of observational cohort data.

#### Randomised controlled trials within the cohort

The BiBBS cohort will form a platform to assess the effectiveness of a selection of BSB interventions using a randomised controlled Trials within Cohorts design (TwiCs; also called the cohort multiple randomized controlled trial design) [15]. TwiCs are randomised controlled trials that are implemented within cohort study samples, with regular outcome measurement as part of the cohort data collection. BiBBS participants will be asked to provide consent to be part of a TwiCs study during cohort recruitment. In this case, routinely collected health record data will be used as outcome measurements.

Eligible participants for each intervention chosen for inclusion in a TwiCs evaluation will be identified from the cohort sample. A group will be randomly selected to receive the intervention, and their outcomes will be compared with eligible participants who were not randomly selected. The process can be repeated many times within a cohort, such that a cohort study hosts multiple TwiCs [15].

It is planned that the cohort will host at least three TwiCs evaluations. A rapid consensus exercise has been conducted to identify possible interventions to undergo TwiCs evaluation, based on the current evidence base (i.e. filling a need for generating evidence and not duplicating existing evidence), and on ethical and logistical grounds. Interventions commissioned by BSB may not be withheld from families; however capacity issues may result in some families not receiving an intervention or having to wait to take part. In this context, random selection provides an ethical approach to selecting who takes part. Final decision on eligibility for TwiCs will be made once interventions have been implemented and capacity issues have been assessed. Separate protocols will be prepared for each TwiCs evaluation.

##### Quasi-experimental design

For most of the BSB interventions, random allocation of families will not be possible, due to ethical and logistical constraints. Quasi-experimental methods will be employed to estimate the causal effects of these BSB interventions. We will consider a range of methods, including propensity scores, regression discontinuity and instrumental variables. Quasi-experimental methods are recommended to evaluate interventions or policy changes in ‘real-world’ circumstances where researchers are not able to manipulate which families receive an intervention [12].

Propensity score approaches can be employed for all interventions that have been taken up by a group within the cohort in order to weight or match a balanced control group. Propensity scores (representing the predicted probability that an individual or family will take part in an intervention, given their baseline characteristics) will be calculated using data collected at baseline. The outcomes for the two groups can then be compared. This approach will allow selection bias to be minimised in the analysis, and the causal effects of interventions to be inferred [23, 24]. For example, for participants of an intervention to support women with mental health problems, propensity scores could be calculated to match a control group of women with similar baseline characteristics including depression and anxiety screening scores, socioeconomic status and ethnicity. Outcomes for women who have taken part in the intervention can then be compared with the matched control group to estimate the intervention effects, for example on maternal mental health, mother-child attachment and child development. However, where factors that predict take-up of interventions have not been identified or accurately measured at baseline, there will be residual differences between groups and remaining concerns about selection biases.

Regression discontinuity designs can be used for projects that have an eligibility cut-off for individuals/families, based on a continuous assignment variable (e.g. low BMI, age 19 or lower) [25, 26]. Regression discontinuity approaches will model child outcomes on the assignment variable, to assess whether there is a gap in outcomes (discontinuity) at the eligibility cut-off. Where possible, analysis will be restricted to families/individuals whose assignment variable scores are close to the cut-off, where the intervention can be thought of as randomly assigned (especially if there is measurement error for the continuous variable). The difference in mean outcomes in the groups just above and below the cut-off is the average causal effect. For example, for an intervention targeting teenage pregnancies age at conception can be used as the assignment variable. Outcomes for women either side of the cut off (i.e. age 20) can be compared to estimate the effect of the intervention, for example on breastfeeding initiation or mother-child attachment.

##### Analysing the Effect of Stacked Interventions

We will consider both the effects of single interventions, and the cumulative effects of stacked (multiple) interventions for pregnant women and children in early life. Pathways through the 22 interventions will be identified for target groups (e.g. pregnant teenagers, women with mental health issues, women with no English language skills) and key outcome domains (see Table 3). We will analyse the effect of attending a single intervention and of attending a pathway of stacked interventions. The BSB programme offers a unique opportunity to complete a novel and pragmatic analysis of the impact of stacked interventions for at risk groups of mothers and children.

**Table 3:** Additional measurements collected from participants

	Mother		Partner	Baby
	Recruitment	Birth	Recruitment	Birth
Baseline Questionnaire	x		x	
Biological Samples				
Blood	x			
Urine	x			
Cord Blood				x
Saliva			x	
Hair		x		
Anthropometry				
Weight	x		x	x
Height	x		x	
Mid Upper Arm circumference	x			
Triceps skinfold thickness	x			x
Subscapular skinfold thickness				x
Abdominal circumference			x	x
Other Measures				
Carotenoids	x			

##### Process Evaluation / Monitoring and Fidelity

The cohort will also facilitate on-going quality improvement for BSB interventions. On-going measurement and monitoring of intervention uptake and outcomes will enable learning and adaptation of interventions to ensure that they are widely used and effective. A separate protocol will be written for the process evaluation, which will follow Medical Research Council (MRC) guidance [27], incorporating the Conceptual Framework for Implementation Fidelity [28].

### Project management

The success of BiBBS is dependent upon input from a wide range of people, organisations and academic partners. The cohort will be managed and monitored using the same management structure established for the BiB cohort [16] and the groups established for BSB (e.g. Partnership board and the CRAG). Full details of the management structure can be seen in Additional file 1.

### Data sharing

The BiBBS experimental cohort study will enable the evaluation of the BSB interventions and increase understanding about how to improve health and development in inner city Bradford. It will contribute knowledge and understanding about the effects of prenatal and child interventions, and the causes of disease that will have international relevance. The BiBBS cohort also offers a platform to develop and test new early childhood interventions, and conduct research projects in a diverse population. Researchers with relevant proposals are encouraged to visit the BiB website (www.borninbradford.nhs.uk) to find out more about the application and selection process.

In addition, data and samples collected throughout the course of the cohort will be available to external researchers and proposals for collaboration will be welcomed. Until recruitment is complete, and all routine outcome measures have been linked and data cleaned, such access may be limited. Researchers with proposals to use the cohort data should complete an outline data request pro forma available on the BiB website (www.borninbradford.nhs.uk).

## Discussion

The BiBBS study builds on the success and momentum of the BiB birth cohort study which recruited over 13,500 babies born across the city between 2007 and 2011 and has developed internationally-leading experience in the administration and collection of data and community relationships [14, 16].

Birth cohorts are prospective, longitudinal epidemiological studies which provide some of the most robust evidence of associations between early life exposures and later outcomes of health and well-being. They can help identify modifiable risk factors that can be targeted in subsequent interventions. However they have not previously been set up to test whether interventions are effective. The focus of the BiBBS cohort is on intervention rather than observation. As far as we are aware, no other birth cohorts have used this design, making BiBBS the world’s first such experimental birth cohort study.

Many risk factors for adverse health and well-being outcomes in children are already well described: parental lifestyle factors such as smoking; child lifestyle factors such as diet and physical activity; family functioning such as parenting practices; health service access; environmental factors such as air pollution [4, 5]. The crucial research goal is to find better ways of changing behaviours and environments to promote positive health and protect against harmful exposures.

BSB provides a £49 million natural experiment to inform public health policy and practice. Our novel experimental birth cohort design has the potential to help provide timely evidence of effectiveness of multiple interventions, a situation that has changed little in the 14 years since the Wanless report identified an “almost complete lack of an evidence base on the cost-effectiveness of public health interventions” [29]. When the proposal for BSB was developed, we were surprised by the poor quality of evidence available for early life interventions. Of the 22 interventions only two were evidence based (tested and proven effective using robust study designs). Since then, one of the two has been found to be ineffective in the UK [30]. This uncertainty highlights the importance of building in robust evaluation to BSB. Over the next 10 years we will be able to address this uncertainty and add to our knowledge base on early life interventions.

The evaluation of complex interventions is challenging. RCTs are considered to be the gold standard, however they are not always feasible or ethical, are time consuming and expensive, and findings don’t always translate across different contexts. For example the Family Nurse Partnership programme had benefits for birth weight, maternal smoking, inter-pregnancy interval and infant hospital admissions in RCTs in the US, but these benefits were not seen in a recent UK trial [30–33]. The natural BSB experiment provides a unique opportunity to evaluate the effects of multiple, individual interventions on child outcomes using TwiCs where it is ethical and feasible to randomly allocate families to interventions, and other approaches, including quasi-experimental methods where interventions are not randomised. The collective effects of stacked interventions on child outcomes will also be assessed. This design will be efficient both in terms of time and costs of evaluation as well as providing a ‘real world’ evaluation of the cumulative effect of multiple interventions. The use of routine health and education data linkage will provide large scale, efficient and timely outcomes that are relevant to practice and policy.

We acknowledge the uncertainties associated with this experimental cohort design. The commissioning and design of the interventions are out of our control, and our key challenge is to be flexible and adapt our choice of evaluation methods to the interventions as they are designed. The evaluations can only be developed as each intervention is designed and we plan an iterative process for this. We recognise potential limitations in internal validity, including identifying a comparable control group, and our aim is to minimise these biases through collection of detailed baseline data and use of quasi-experimental methods to estimate causal effects. This efficient evaluation of interventions as they are implemented within the BSB programme will support the understanding of effectiveness of multiple interventions. The evidence of effectiveness of these interventions is likely to be generalizable to other deprived, multi-ethnic urban populations.

Over the last 10 years we have developed considerable expertise in the birth cohort design, implementation and analysis with BiB. A key reflection over this period is that while the risk factors for adverse health and social outcomes are increasingly well described, the solutions to tackling them remain as elusive as ever. The BiBBS experimental birth cohort study can contribute much needed evidence to inform policy makers and practitioners about effective approaches to improve health and well-being for future generations.

## Abbreviations

BiB, Born in Bradford; BiBBS, Born in Bradford’s Better Start; BSB, Better Start Bradford; BTHFT, Bradford Teaching Hospitals NHS Foundation Trust; CRAG, Community Representatives Advisory Group; GCP, Good Clinical Practice; GTT, Glucose Tolerance Test; MRC, Medical Research Council; RCT, Randomised Controlled Trial; TwiCs, Trials within Cohorts; UPN, Unique Pupil Number

## Additional file

Additional file 1: Additional information on study methods and management. (DOC 1533 kb)

## References

[1] Annual Report of the Chief Medical Officer (2012). Our children deserve better: prevention pays.

[2] Tickell D (2011). The Early Years: Foundations for life, health and learning - An independent report on the Early Years Foundation Stage to Her Majesty’s Government.

[3] Allen G (2011). Early intervention: The next steps - an independent report to Her Majesty’s Government.

[4] WHO Commission on Social Determinants of Health (2008). Closing the gap in a generation: health equity through action on the social determinants of health: commission on social determinants of health final report.

[5] Marmot M, Allen J, Goldblatt P, Boyce T, McNeish D, Grady M, Geddes I (2010). Fair society, healthy lives: strategic review of health inequalities in England post 2010.

[6] Axford N, Barlow J (2013). What works: An overview of the best available evidence on giving children a better start.

[7] Guyatt GH, Sackett DL, Sinclair JC, Hayward R, Cook DJ, Cook RJ (1995). Users’ guides to the medical literature. IX. A method for grading health care recommendations. Evidence-Based Medicine Working Group. JAMA.

[8] Prescott RJ, Counsell CE, Gillespie WJ, Grant AM, Russell IT, Kiauka S, Colthart IR, Ross S, Shepherd SM, Russell D (1999). Factors that limit the quality, number and progress of randomised controlled trials. HTA.

[9] Datta J, Petticrew M (2013). Challenges to evaluating complex interventions: a content analysis of published papers. BMC Public Health.

[10] Craig P, Dieppe P, Macintyre S, Michie S, Nazareth I, Petticrew M (2013). Developing and evaluating complex interventions: the new Medical Research Council guidance. Int J Nurs Stud.

[11] Petticrew M, Cummins S, Ferrell C, Findlay A, Higgins C, Hoy C, Kearns A, Sparks L (2005). Natural experiments: an underused tool for public health?. Public Health.

[12] Craig P, Cooper C, Gunnell D, Haw S, Lawson K, Macintyre S, Ogilvie D, Petticrew M, Reeves B, Sutton M (2012). Using natural experiments to evaluate population health interventions: new MRC guidance. J Epidemiol Community Health.

[13] Raghupathi W, Raghupathi V (2014). Big data analytics in healthcare: promise and potential. Health Inf Sci Syst.

[14] Wright J, Small N, Raynor P, Tuffnell D, Bhopal R, Cameron N, Fairley L, Lawlor DA, Parslow R, Petherick ES (2013). Cohort profile: The Born in Bradford multi-ethnic family cohort study. Int J Epidemiol.

[15] Relton C, Torgerson D, O’Cathain A, Nicholl J. Rethinking pragmatic randomised controlled trials: introducing the “cohort multiple randomised controlled trial” design. BMJ. 2010;340. 10.1136/bmj.c1066 20304934

[16] Raynor P, Born in Bradford Collaborative G (2008). Born in Bradford, a cohort study of babies born in Bradford, and their parents: protocol for the recruitment phase. BMC Public Health.

[17] Gov.uk Official Statistics: English indices of deprivation +; File 5: scores for the indices of deprivation, File 6: population denominators. Files combined to derive population weighted IMD scores by region. Source: https://www.gov.uk/government/statistics/english-indices-of-deprivation-2015 Accessed 22/06/2016

[18] Born in Bradford [www.borninbradford.nhs.uk]

[19] Flesch R (1948). A new readability yardstick. J Appl Psychol.

[20] (BAILII) BaILII: Gillick v West Norfolk & Wisbech Area Health Authority. In: *UKHL 7.* 1985. 11648530

[21] Johnston C, Liddle J (2007). The Mental Capacity Act 2005: a new framework for healthcare decision making. J Med Ethics.

[22] Parliament of the United Kingdom of Great Britain and Northern Ireland (1998). Data protection act.

[23] West SG, Duan N, Pequegnat W, Gaist P, Des Jarlais DC, Holtgrave D, Szapocznik J, Fishbein M, Rapkin B, Clatts M (2008). Alternatives to the randomized controlled trial. Am J Public Health.

[24] Austin PC (2011). An introduction to propensity score methods for reducing the effects of confounding in observational studies. Multivariate Behav Res.

[25] Bor J, Moscoe E, Mutevedzi P, Newell M-L, Bärnighausen T (2014). Regression discontinuity designs in epidemiology: causal inference without randomized trials. Epidemiol.

[26] Moscoe E, Bor J, Bärnighausen T (2015). Regression discontinuity designs are underutilized in medicine, epidemiology, and public health: a review of current and best practice. J Clin Epidemiol.

[27] Moore GF, Audrey S, Barker M, Bond L, Bonell C, Hardeman W, Moore L, O’Cathain A, Tinati T, Wight D (2015). Process evaluation of complex interventions: Medical Research Council guidance. BMJ.

[28] Hasson H (2010). Systematic evaluation of implementation fidelity of complex interventions in health and social care. Implementation Sci.

[29] Wanless D (2004). Securing good health for the whole population. Final Report.

[30] Robling M, Bekkers M-J, Bell K, Butler CC, Cannings-John R, Channon S, Martin BC, Gregory JW, Hood K, Kemp A (2016). Effectiveness of a nurse-led intensive home-visitation programme for first-time teenage mothers (Building Blocks): a pragmatic randomised controlled trial. Lancet.

[31] Olds DL, Henderson CR, Chamberlin R, Tatelbaum R (1986). Preventing child abuse and neglect: a randomized trial of nurse home visitation. Pediatrics.

[32] Kitzman H, Olds DL, Henderson CR, Hanks C, Cole R, Tatelbaum R, McConnochie KM, Sidora K, Luckey DW, Shaver D (1997). Effect of prenatal and infancy home visitation by nurses on pregnancy outcomes, childhood injuries, and repeated childbearing. A randomized controlled trial. JAMA.

[33] Olds DL, Robinson J, O’Brien R, Luckey DW, Pettitt LM, Henderson CR, Ng RK, Sheff KL, Korfmacher J, Hiatt S (2002). Home visiting by paraprofessionals and by nurses: a randomized, controlled trial. Pediatrics.

[34] Office for National Statistics: Ward Level Mid-Year Population Estimates (experimental) 2014; source: https://www.ons.gov.uk/peoplepopulationandcommunity/populationandmigration/populationestimates/datasets/wardlevelmidyearpopulationestimatesexperimental Dataset used: Mid-2014: SAPE17DT8; accessed: 22/04/2016.

[35] Office for National Statistics: Birth Summary Tables - England and Wales; source: http://www.ons.gov.uk/peoplepopulationandcommunity/birthsdeathsandmarriages/livebirths/datasets/birthsummarytables Dataset used: 2014; accessed: 22/04/2016

[36] Office for National Statistics, 2011 Census: Neighbourhood statistics, Ethnic Group, 2011 (KS201EW). Aggregated to derive ‘Better Start Bradford’; sources: http://www.neighbourhood.statistics.gov.uk/dissemination/LeadTableView.do?a=7&b=13689850&c=bowling+and+barkerend&d=14&e=62&g=6369228&i=1001x1003x1032x1004&o=362&m=0&r=1&s=1464882068594&enc=1&dsFamilyId=2477. http://www.neighbourhood.statistics.gov.uk/dissemination/LeadTableView.do?a=7&b=13689851&c=bradford+moor&d=14&e=62&g=6369232&i=1001x1003x1032x1004&o=362&m=0&r=1&s=1464882041781&enc=1&dsFamilyId=2477. http://www.neighbourhood.statistics.gov.uk/dissemination/LeadTableView.do?a=7&b=13689863&c=little+horton&d=14&e=62&g=6369257&i=1001x1003x1032x1004&o=362&m=0&r=1&s=1464881977688&enc=1&dsFamilyId=2477. Accessed: 22/04/2016

[37] Office for National Statistics: Births and infant deaths, England: 2012 to 2014; source: https://www.ons.gov.uk/peoplepopulationandcommunity/birthsdeathsandmarriages/deaths/adhocs/005621birthsandinfantdeathsengland2012to2014 Data used: 2014 Live births, 2014 Deaths Infant, derived rate per 1000 live births; accessed: 22/04/2016

[38] Public Health England: National Dental Epidemiology Programme for England: oral health survey of five-year-old children 2012. A report on the prevalence and severity of dental decay. London; 2012 (Appendix 1): source: http://www.nwph.net/dentalhealth/survey-results5.aspx?id=1 Accessed: 22/04/2016;

[39] Public Health England: Electoral Ward and MSOA NCMP child obesity prevalence; source: http://www.noo.org.uk/visualisation Dataset used: Ward and MSOA obesity prevalence data – NCMP 2011/12 to 2013/14; accessed: 22/04/2016

[40] Health & social Care Information Centre: National Child Measurement Programme - England, 2014-15; source: http://www.hscic.gov.uk/searchcatalogue?productid=19405&q=title%3a%22national+child+measurement+programme%22&sort=Relevance&size=10&page=1#top Dataset used: National Child Measurement Programme – England 2014-15: Tables [.xlsx]; accessed: 22/04/2016

