# Supplementary material for: Born in Bradford’s Better Start: an experimental birth cohort study to evaluate the impact of early life interventions
Source: BMC Public Health. 2016 Aug 4;16(1):711. doi: 10.1186/s12889-016-3318-0 (PMC4996273; doi:10.1186/s12889-016-3318-0)
Supplement: Additional file 1: — Additional information on study methods and management. (DOC 1533 kb) [file 12889_2016_3318_MOESM1_ESM.doc]

**Additional file 1**

**Appendix 1: Translation process for BSB materials – translation to Urdu**

1

Questionnaire translated into Urdu

Back translation in English (independent translator) Feedback to BiBBS Team


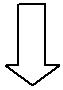


2

First Urdu transliteration


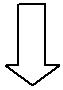


3

Pilot with Urdu bilingual groups Feedback to translators

Second draft of Urdu transliteration compiled Second draft piloted with Urdu monolingual groups Feedback to translators


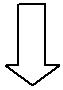


4

Second draft of Urdu transliteration used to compile first Mirpuri transliteration First Mirpuri transliteration compiled


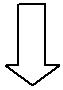


5

First Mirpuri transliteration piloted with Mirpuri bilingual groups Feedback to translators

Second draft of Mirpuri transliteration compiled Second draft piloted with Mirpuri monolingual groups Feedback to translators


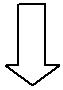


6

Final Urdu and Mirpuri versions completed

**Appendix 2: Baseline Questionnaire for Pregnant Women**

**
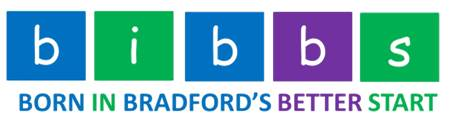
**

**BiBBS questionnaire for pregnant women**

This questionnaire is for pregnant women in the areas Little Horton, Bowling & Barkerend and Bradford Moor. It usually takes about 45 minutes to complete.

This questionnaire is about you and your baby. We are interested to know about your family life and relationships, your house and neighbourhood, the languages you speak, your social and financial circumstances, your health and wellbeing, this pregnancy and your plans for the baby.

We would be grateful if you help us by answering as many of these questions as possible but if there are any questions you do not want to answer that is fine. There are no right or wrong answers.

All the answers you give are confidential. Your name and full address will not appear anywhere on the questionnaire.

We apologise if any questions cause offence – this is not our intention. We are asking everyone the same questions but we realise you may find some questions odd or unusual.

We will start by taking some measurements of your height, weight and arm size.

Thank you for being a part of our BIBBS study!


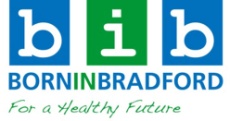

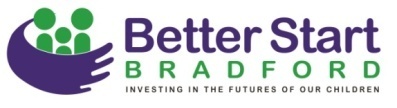

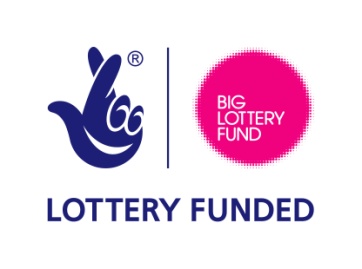


**FRONT SHEET**

**-------------------------------------- TO BE COMPLETED BY RESEARCHER --------------------------------------**

PARTICIPANT INFORMATION

Participant Study ID _______________________________________________

Who is administering the questionnaire? ______________________________________________

Date completing questionnaire   

Language used for administration  English

 Punjabi

 Urdu

 Other: ____________

Interpreter used  No

 Yes, family or friend

 Yes, other

Who is present at the interview  Partner

 Family member

 Friend

 Other

 None

Gestational age at completion  weeks  Don’t know

MEASUREMENTS

Who is taking the measurements? __________________________________________

Height   cms  Not able to take
Weight   kgs  Not able to take

Triceps   cms  Not able to take

**SECTION A BACKGROUND**

*This first section is about your background.*

18. What is your date of birth?

  

Day Month Year

19. What country were you born in?

 England  Northern Ireland  Poland

 Pakistan  Scotland  Czech Republic

 Bangladesh  Wales  Slovakia

 India  Republic of Ireland  Romania

 Hungary

 Other: ________________________

20. What country were your parents born in?

Your mother: Your father:

  England

  Pakistan

  Bangladesh

  India

  Northern Ireland

  Scotland

  Wales

  Republic of Ireland

  Slovakia

  Czech Republic

  Poland

  Romania

  Hungary

  Other: __________________________________________________

  Don’t know

22. If you were not born in the UK, how old were you when you moved to the UK?

 years old  Don’t know

24. What best describes your ethnic group or background?

- White; English/Welsh/Scottish/Northern Irish/British
- White; Irish
- Pakistani
- Indian
- Bangladeshi
- White; Polish
- White; Slovakian
- White; Romanian
- White; Czech
- Other White
- White; Gypsy/Roma or Irish traveller
- Chinese
- African
- Caribbean
- Mixed White and Black Caribbean
- Mixed White and Black African
- Mixed White and Asian
- Any other mixed/multiple ethnic background: ________________________________________
- Any other ethnic background: ________________________________________

25. What is your religion?

- None
- Christian (including Church of England, Catholic, Protestant and all Christian denominations)
- Islam
- Sikhism
- Buddhism
- Hinduism
- Judaism
- Any other religion: ________________________________________

26. How often, if at all, do you pray, or attend services or meetings connected with your religion?

- Every day or more
- Not every day but at least once a week
- Less often than once a week but at least once a month
- Sometimes but less than once a month
- Very rarely or never

**SECTION B HOUSEHOLD INFORMATION**

*This section is about the people in your household and their relationships. If you are unsure please ask the researcher – he or she can help you with these questions.*

27. How many people live in your household, including yourself?  people

28-69. For the other members of your household, what is their age, sex and relationship **to the baby**?

| **No.** | Age | Sex | Relationship to baby |
| --- | --- | --- | --- |
| **1** |  years old  or if less than 1 year:   months old   Don’t know |  Male   Female | - Natural father, - Adoptive mother/father, - Foster mother/father, - Stepmother/father, - Partner of mother/father - Grandmother/grandfather, - Aunt/uncle - Brother or sister (same mother and father) - Half-brother or sister (same mother or father but not both) - Step-brother or sister (different mother and father) - Cousin - Other: __________________________________ |
| **2** |  years old  or if less than 1 year:   months old   Don’t know |  Male   Female | - Natural father, - Adoptive mother/father, - Foster mother/father, - Stepmother/father, - Partner of mother/father - Grandmother/grandfather, - Aunt/uncle - Brother or sister (same mother and father) - Half-brother or sister (same mother or father but not both) - Step-brother or sister (different mother and father) - Cousin - Other: __________________________________ |
| **3** |  years old  or if less than 1 year:   months old   Don’t know |  Male   Female | - Natural father, - Adoptive mother/father, - Foster mother/father, - Stepmother/father, - Partner of mother/father - Grandmother/grandfather, - Aunt/uncle - Brother or sister (same mother and father) - Half-brother or sister (same mother or father but not both) - Step-brother or sister (different mother and father) - Cousin - Other: __________________________________ |
| **4** |  years old  or if less than 1 year:   months old   Don’t know |  Male   Female | - Natural father, - Adoptive mother/father, - Foster mother/father, - Stepmother/father, - Partner of mother/father - Grandmother/grandfather, - Aunt/uncle - Brother or sister (same mother and father) - Half-brother or sister (same mother or father but not both) - Step-brother or sister (different mother and father) - Cousin - Other: __________________________________ |
| **5** |  years old  or if less than 1 year:   months old   Don’t know |  Male   Female | - Natural father, - Adoptive mother/father, - Foster mother/father, - Stepmother/father, - Partner of mother/father - Grandmother/grandfather, - Aunt/uncle - Brother or sister (same mother and father) - Half-brother or sister (same mother or father but not both) - Step-brother or sister (different mother and father) - Cousin - Other : __________________________________ |
| **6** |  years old  or if less than 1 year:   months old   Don’t know |  Male   Female | - Natural father, - Adoptive mother/father, - Foster mother/father, - Stepmother/father, - Partner of mother/father - Grandmother/grandfather, - Aunt/uncle - Brother or sister (same mother and father) - Half-brother or sister (same mother or father but not both) - Step-brother or sister (different mother and father) - Cousin - Other: __________________________________ |
| **7** |  years old  or if less than 1 year:   months old   Don’t know |  Male   Female | - Natural father, - Adoptive mother/father, - Foster mother/father, - Stepmother/father, - Partner of mother/father - Grandmother/grandfather, - Aunt/uncle - Brother or sister (same mother and father) - Half-brother or sister (same mother or father but not both) - Step-brother or sister (different mother and father) - Cousin - Other: __________________________________ |
| **8** |  years old  or if less than 1 year:   months old   Don’t know |  Male   Female | - Natural father, - Adoptive mother/father, - Foster mother/father, - Stepmother/father, - Partner of mother/father - Grandmother/grandfather, - Aunt/uncle - Brother or sister (same mother and father) - Half-brother or sister (same mother or father but not both) - Step-brother or sister (different mother and father) - Cousin - Other: __________________________________ |
| **9** |  years old  or if less than 1 year:   months old   Don’t know |  Male   Female | - Natural father, - Adoptive mother/father, - Foster mother/father, - Stepmother/father, - Partner of mother/father - Grandmother/grandfather, - Aunt/uncle - Brother or sister (same mother and father) - Half-brother or sister (same mother or father but not both) - Step-brother or sister (different mother and father) - Cousin - Other: __________________________________ |
| **10** |  years old  or if less than 1 year:   months old   Don’t know |  Male   Female | - Natural father, - Adoptive mother/father, - Foster mother/father, - Stepmother/father, - Partner of mother/father - Grandmother/grandfather, - Aunt/uncle - Brother or sister (same mother and father) - Half-brother or sister (same mother or father but not both) - Step-brother or sister (different mother and father) - Cousin - Other: __________________________________ |
| **11** |  years old  or if less than 1 year:   months old   Don’t know |  Male   Female | - Natural father, - Adoptive mother/father, - Foster mother/father, - Stepmother/father, - Partner of mother/father - Grandmother/grandfather, - Aunt/uncle - Brother or sister (same mother and father) - Half-brother or sister (same mother or father but not both) - Step-brother or sister (different mother and father) - Cousin - Other: __________________________________ |
| **12** |  years old  or if less than 1 year:   months old   Don’t know |  Male   Female | - Natural father, - Adoptive mother/father, - Foster mother/father, - Stepmother/father, - Partner of mother/father - Grandmother/grandfather, - Aunt/uncle - Brother or sister (same mother and father) - Half-brother or sister (same mother or father but not both) - Step-brother or sister (different mother and father) - Cousin - Other (clarify): ______________________________ |

73-103. If your baby has any brothers or sisters not living in the same household, what are their ages, sex and relationship **to the baby**?

| **No.** | Age | Sex | Relationship to baby |
| --- | --- | --- | --- |
| **1** |  years old  or if less than 1 year:   months old   Don’t know |  Male   Female | - Brother or sister (same mother and father) - Half-brother or sister (same mother or father but not both) - Step-brother or sister (different mother and father) |
| **2** |  years old  or if less than 1 year:   months old   Don’t know |  Male   Female | - Brother or sister (same mother and father) - Half-brother or sister (same mother or father but not both) - Step-brother or sister (different mother and father) |
| **3** |  years old  or if less than 1 year:   months old   Don’t know |  Male   Female | - Brother or sister (same mother and father) - Half-brother or sister (same mother or father but not both) - Step-brother or sister (different mother and father) |

104. What is your relationship with the baby’s natural father?

- Married to baby’s father
- In a relationship with baby’s father but not married
- Separated or divorced
- Never been in a relationship with baby’s father
- Baby’s father has died

105. Are you living with the baby’s natural father?

- Yes
- No

107. If you have a partner at the moment, what is your partner’s date of birth?

    Don’t know

Day Month Year

108. Which country was the father of your baby born in?

🞏 England 🞏 Northern Ireland 🞏 Slovakia

🞏 Pakistan 🞏 Scotland 🞏 Czech Republic

🞏 Bangladesh 🞏 Channel Islands 🞏 Poland

🞏 India 🞏 Isle of Man 🞏 Philippines

🞏 Republic of Ireland 🞏 Other: _________________________

🞏 Don’t know

109. Are you related to the father of your baby other than by marriage?
*If you are unsure please ask the researcher – he or she can help you with this question.*

🞏 Yes

🞏 No

🞏 Don’t know

🞏 Do not wish to answer

110. If yes, how are you related to the father of your baby?
*If you are unsure please ask the researcher – he or she can help you with this question.*

🞏 First cousin

🞏 First cousin, once removed

🞏 Second cousin

🞏 Other related by blood

🞏 Don’t know

**SECTION C HOUSE**

*This next section is about the house you live in at the moment and your previous home.*

111. What is your postcode?  

112. How long have you lived at your current address?

 years and  months

113. Is your home:

- a house or bungalow
- a flat or maisonette
- a studio flat
- a bedsit or room in a house
- another type of home
- don’t know

114. How many bedrooms does your household have, including spare bedrooms?

 bedrooms

115. Do you (and/or your partner) own or rent your home, or have some other arrangement?

- own with help of mortgage or loan
- own outright
- rent it
- live here rent free (including rent free in relatives’/friends’ property but not squatting)
- living in a relatives’/friends’ property and paying board
- pay rent and part mortgage (shared ownership)
- squatting
- don’t know

116. If you are renting your home, who is your landlord?

- Private landlord or letting agency
- Housing association, housing co-operative, charitable trust
- Local authority, local council
- Relative or friend (before you lived here) of a household member
- Employer (individual) of a household member
- Employer (company) of a household member
- Another organisation
- Don’t know

117. What was the main reason you moved from your last home?

- Needing a bigger/better home or a home in a better area
- Went from a rented home to buying a home
- Relationship breakdown
- Moving in with partner and/or moving out of parents’ home
- To live closer to my/my partner’s work
- Wanted to live closer to friends/relatives
- Moved to Bradford from abroad
- End of rental agreement last property (contract was up)
- Issues/arguments with previous landlord
- Issues/arguments with neighbours
- Issues/arguments with family/partner/ housemates living in previous home
- Other
- Not applicable; never moved from first home

118. What is the postcode of your previous home?

 

- Not applicable; moved here from abroad
- Don’t know

119. How often did you move in the past 5 years?

 times

120. Are you planning to move house in the next year?

- Yes
- No
- Don’t know

**SECTION D NEIGHBOURHOOD**

*This next section is about the neighbourhood you live in.*

121. How satisfied or dissatisfied are you with the area you live in?
*By your area, I mean within about a mile or 20 minute walk of your home.*

- Very satisfied
- Fairly satisfied
- Neither satisfied nor dissatisfied
- Fairly dissatisfied
- Very dissatisfied

122. How satisfied or dissatisfied are you with the parks and green spaces in your local area?

*By your area, I mean within about a mile or 20 minute walk of your home.*

- Very satisfied
- Fairly satisfied
- Neither satisfied nor dissatisfied
- Fairly dissatisfied
- Very dissatisfied

123-124. How often do you visit Bradford’s parks and green spaces?

During the winter months (September – March) During spring and summer (April - August)

- 5 times a week or more  5 times a week or more
- 2 to 4 times a week  2 to 4 times a week
- once a week  once a week
- 1 to 3 times a month  1 to 3 times a month
- less than once a month  less than once a month

125. Thinking about your neighbourhood, to what extent do you agree with this statement:

*Other people think this is a good area.*

- Strongly agree
- Agree
- Neither agree nor disagree
- Disagree
- Strongly disagree

126. Do children in your area have an outdoor space or facilities nearby where they can play safely?

- Yes
- No
- Don’t know

**SECTION E LANGUAGE**

*This next section is about languages you speak, languages in your households and books in your home.*

127. What is your first language?

*Please tick two boxes if you are bilingual (you speak two languages fluently and were taught these languages from a young age).*

- English
- Punjabi
- Urdu (including Hindi)
- Polish
- Slovakian
- Hungarian
- Romanian
- Russian
- Gujarati
- Spanish
- Pashto
- Bengali (including Sylheti, Chatgaya/Chittagonian)
- Arabic
- Other: ______________________

128. Which of these languages are usually spoken at home?

- English
- Punjabi
- Urdu (including Hindi)
- Polish
- Slovakian
- Hungarian
- Romanian
- Russian
- Gujarati
- Spanish
- Pashto
- Bengali (including Sylheti, Chatgaya/Chittagonian)
- Arabic
- Other: ______________________

129. Which of these languages do you speak most often outside the home?

- English
- Punjabi
- Urdu (including Hindi)
- Polish
- Slovakian
- Hungarian
- Romanian
- Russian
- Gujarati
- Spanish
- Pashto
- Bengali (including Sylheti, Chatgaya/Chittagonian)
- Arabic
- Other: ______________________

130. *If you don’t have children yet please skip this question.*If you have children already, which languages do they speak?

- English
- Punjabi
- Urdu (including Hindi)
- Polish
- Slovakian
- Hungarian
- Romanian
- Russian
- Gujarati
- Spanish
- Pashto
- Bengali (including Sylheti, Chatgaya/Chittagonian)
- Arabic
- Other: ______________________

*If English is your first language please skip the next question and go to 132.*

131. If English is not your first language, how well can you do the following things **in English**:

**Not at all A little bit Some Quite well Very well**

Listening     

Reading     

Writing     

Speaking     

132. How well can you do the following things **in your first language**:

*If you are bilingual choose the language you are most comfortable with.*

**Not at all A little bit Some Quite well Very well Does not apply**

Listening     

Reading      

Writing      

Speaking     

133. With which statement do you agree the most? Please tick only one box.

- It is important to me that my child **does** learn English before starting school
- It does not matter to me
- It is important to me that my child **does not** learn English before starting school

134. How many books for **adults** do you have in your home?

- 0-10
- 11-50
- more than 50

135. How often do you read books for pleasure?

- (Almost) never
- Once or twice a month
- About once a week
- Several times a week
- Daily

*If you don’t have children yet please skip the next two questions and go to F1.*

136. If you have children already, how many books for **children** do you have in your home?

- 0-10
- 11-50
- more than 50

137. If you have preschool children or children in primary school already, how many days in a typical week do you read with them?

- 0
- 1
- 2
- 3
- 4
- 5
- 6
- 7

**SECTION F EDUCATION**

*This next section is about you and your partners’ education. Please skip the questions that don’t apply to you. We first ask about your own education.*

138. Where did you obtain your highest educational qualification?

- England
- Pakistan
- India
- Bangladesh
- Poland
- Slovakia
- Hungary
- Elsewhere

139. If your highest educational qualification was obtained in **England**, what is the highest level you completed? *Please tick only one box.*

- Less than 5 GCSEs (A*-C), CSE or O-Levels
- 5 or more GCSEs (A*-C), SCEs or O-Levels
- GNVQ foundation level
- NVQ1
- GNVQ intermediate
- NVQ 2
- (Young) apprenticeship
- NVQ 3
- Advanced apprenticeship
- GNVQ Advanced
- AS or A level, International Baccalaureate or BTEC
- NVQ 4/5
- Higher National Certificate/ Higher National Diploma, Higher Education Diploma
- Foundation Degree
- Bachelor’s degree 3-5 years
- Master’s degree (taught/research) or Postgraduate qualification, Doctorate/ PhD
- Other: _______________________________
- None of these qualifications
- Don’t know

140. If your highest educational qualification was obtained in **Pakistan**, what is the highest level you completed? *Please tick only one box.*

- Secondary School Certificate/ Matriculation
- Higher Secondary or Intermediate Certificate
- Technical Education Certificate/ Diploma
- Registered Nurse/ General Nursing Diploma
- Primary Teaching Certificate
- Certificate in Training, Paramedic Secondary School Certificate/ Matriculation
- Bachelor Degree Arts/Sciences, Education, Engineering, Medicine/Surgery
- Master Degree/ Master of Education/ Master of Philosophy (MPhil), Doctorate
- Other: ________________________________
- None of these qualifications
- Don’t know

141. If your highest educational qualification was obtained in **India**, what is the highest level you completed?

*Please tick only one box.*

- Senior Secondary School Leaving Certificate
- Matriculation Certificate, Senior School Certificate, ITI Certificate
- Diploma in Technical Education, Junior basic teacher’s training, Nursing Diploma/Certificate
- Bachelor’s Degree, Bachelor of Law/ Education
- Master’s Degree, Master of Philosophy (Mphil), Doctor of Philosophy/ Letters
- Other: ________________________________
- None of these qualifications
- Don’t know

142. If your highest educational qualification was obtained in **Bangladesh**, what is the highest level you completed? *Please tick only one box.*

- Secondary School/ Higher Secondary Certificate
- Dakhil/ Alim Certificate
- SSC Vocational/ Trade Certificate
- HSC Business Management
- HSC Vocational
- Diploma in Commerce Certificate
- Diploma in Engineering
- Certificate in Education (CinEd)
- Diploma in Agriculture/Ayurvedic & Unanai Medicine/ Textile/ Health Technology/ Survey/ Ceramics/ Nursing/ Graphic Arts
- Bachelor Degree Pass, Bachelor Degree
- Master’s Degree, PGD and PhD
- Fazil/ Kamil Degree
- Other: ________________________________
- None of these qualifications
- Don’t know

143. If your highest educational qualification was obtained in **Poland**, what is the highest level you completed? *Please tick only one box.*

- General Secondary School Leaving Certificate/ Diploma
- Technikum, Liceum Mature Certificate/Diploma
- Technical Secondary School, Specialised Secondary School
- General/ Supplementary Secondary School
- Ballet School Diploma (technician level), Second level music school Diploma, Circus School Diploma (technician level)
- Basic vocational School
- Post-secondary School Certificate/ Diploma
- Teacher Training, Diploma Social Work College
- 1st Degree Studies
- University Studies, Magister/Lekarz, Postgraduate Certificate
- Master’s Degree, Doctoral Study/PhD
- Other: ________________________________
- None of these qualifications
- Don’t know

144. If your highest educational qualification was obtained in **Slovakia**, what is the highest level you completed? *Please tick only one box.*

- Study of selected subjects (Štúdium jednotlivých predmetov)
- Vocational School (Odborné učilište)
- Secondary Specialised School without maturita (Vysvedčenie o záverečnej skúške, Výučný list)
- Retraining Courses Certificate (Osvedčenie)
- Secondary Specialised School with maturita (Vysvedčenie o maturitnej skúške, Vysvedčenie o maturitnej skúške, Výučný list)
- Gymnasium (gymnázium)
- Follow-up courses (Nadstavbové štúdium)
- Post-secondary Qualification Study (Pomaturitné kvalifikačné štúdium)
- Supplementary Pedagogical Study (Doplňujúce pedagogické štúdium)
- Post-secondary Specialised Study (Pomaturitné špecializačné štúdium)
- Conservatoire (Konzervatórium)
- Higher Professional Studies/ Graduate Diploma (Absolventský diplom), Bachelor
- Master, Magister, Doctoral, Engineer, PhD, Extensive study for teaching diploma
- Other: ________________________________
- None of these qualifications
- Don’t know

145. If your highest educational qualification was obtained in **Hungary**, what is the highest level you completed? *Please tick only one box.*

- Certification of the Maturity Examination (Párhuzamos oktatás szakközépiskoláb)
- Upper Secondary General School
  ( Gimnázium)
- Upper Secondary Vocational School (Szakközépiskola)
- Vocational certificate based on NVQL examination (Párhuzamos oktatás szakiskolában, Szakiskola, előkészítő szakiskola, Alapfokú iskolai végzettségre épülő szakképzés, évfolyamra épülő szakképzés)
- Vocational certificate based on NVQL examination (Szakképző évfolyamok középiskola utolsó évfolyamára vagy érettségire épülő nem felsőfokú OKJ szakmákban)

- Tertiary Vocational Program (Felsőfokú szakképzés)
- Bachelor’s Degree (Alapképzés), Master’s Degree (Osztatlan képzés/ Master), University Diploma (Egyetemi szintű alapképzés), College Diploma (Főiskolai szintű alapképzések), Certificate in Specialisation (Szakirányú továbbképzés), Doctorate/PhD
- Other: _____________________________
- None of these qualifications
- Don’t know

146. If your highest educational qualification was obtained elsewhere, what is the highest level you completed?

Country: _______________________________________________________________

Type of school/institution: _______________________________________________________________

Educational programme: _______________________________________________________________

Degree/qualification: _______________________________________________________________

- Don’t know

*The next few questions are about the education of your partner.*

147. Where did your partner obtain his/her highest educational qualification?

- England
- Pakistan
- India
- Bangladesh
- Poland
- Slovakia
- Hungary
- Elsewhere
- Don’t know

148. If your partner’s highest educational qualification was obtained in **England**, what is the highest level he/she completed? *Please tick only one box.*

- Less than 5 GCSEs (A*-C), CSE or O-Levels
- 5 or more GCSEs (A*-C), SCEs or O-Levels
- GNVQ foundation level
- NVQ1
- GNVQ intermediate
- NVQ 2
- (Young) apprenticeship
- NVQ 3
- Advanced apprenticeship
- GNVQ Advanced
- AS or A level, International Baccalaureate or BTEC
- NVQ 4/5
- Higher National Certificate/ Higher National Diploma, Higher Education Diploma
- Foundation Degree
- Bachelor’s degree 3-5 years
- Master’s degree (taught/research) or Postgraduate qualification, Doctorate/ PhD
- Other: _______________________________
- None of these qualifications
- Don’t know

149. If your partner’s highest educational qualification was obtained in **Pakistan**, what is the highest level he/she completed? *Please tick only one box.*

- Secondary School Certificate/ Matriculation
- Higher Secondary or Intermediate Certificate
- Technical Education Certificate/ Diploma
- Registered Nurse/ General Nursing Diploma
- Primary Teaching Certificate
- Certificate in Training, Paramedic Secondary School Certificate/ Matriculation
- Bachelor Degree Arts/Sciences, Education, Engineering, Medicine/Surgery
- Master Degree/ Master of Education/ Master of Philosophy (MPhil), Doctorate
- Other: ________________________________
- None of these qualifications
- Don’t know

150. If your partner’s highest educational qualification was obtained in **India**, what is the highest level he/she completed?

*Please tick only one box.*

- Senior Secondary School Leaving Certificate
- Matriculation Certificate, Senior School Certificate, ITI Certificate
- Diploma in Technical Education, Junior basic teacher’s training, Nursing Diploma/Certificate
- Bachelor’s Degree, Bachelor of Law/ Education
- Master’s Degree, Master of Philosophy (Mphil), Doctor of Philosophy/ Letters
- Other: ________________________________
- None of these qualifications
- Don’t know

151. If your partner’s highest educational qualification was obtained in **Bangladesh**, what is the highest level he/she completed? *Please tick only one box.*

- Secondary School/ Higher Secondary Certificate
- Dakhil/ Alim Certificate
- SSC Vocational/ Trade Certificate
- HSC Business Management
- HSC Vocational
- Diploma in Commerce Certificate
- Diploma in Engineering
- Certificate in Education (CinEd)
- Diploma in Agriculture/Ayurvedic & Unanai Medicine/ Textile/ Health Technology/ Survey/ Ceramics/ Nursing/ Graphic Arts
- Bachelor Degree Pass, Bachelor Degree
- Master’s Degree, PGD and PhD
- Fazil/ Kamil Degree
- Other: ________________________________
- None of these qualifications
- Don’t know

152. If your partner’s highest educational qualification was obtained in **Poland**, what is the highest level he/she completed? *Please tick only one box.*

- General Secondary School Leaving Certificate/ Diploma
- Technikum, Liceum Mature Certificate/Diploma
- Technical Secondary School, Specialised Secondary School
- General/ Supplementary Secondary School
- Ballet School Diploma (technician level), Second level music school Diploma, Circus School Diploma (technician level)
- Basic vocational School
- Post-secondary School Certificate/ Diploma
- Teacher Training, Diploma Social Work College
- 1st Degree Studies
- University Studies, Magister/Lekarz, Postgraduate Certificate
- Master’s Degree, Doctoral Study/PhD
- Other: ________________________________
- None of these qualifications
- Don’t know

153. If your partner’s highest educational qualification was obtained in **Slovakia**, what is the highest level he/she completed? *Please tick only one box.*

- Study of selected subjects (Štúdium jednotlivých predmetov)
- Vocational School (Odborné učilište)
- Secondary Specialised School without maturita (Vysvedčenie o záverečnej skúške, Výučný list)
- Retraining Courses Certificate (Osvedčenie)
- Secondary Specialised School with maturita (Vysvedčenie o maturitnej skúške, Vysvedčenie o maturitnej skúške, Výučný list)
- Gymnasium (gymnázium)
- Follow-up courses (Nadstavbové štúdium)
- Post-secondary Qualification Study (Pomaturitné kvalifikačné štúdium)
- Supplementary Pedagogical Study (Doplňujúce pedagogické štúdium)
- Post-secondary Specialised Study (Pomaturitné špecializačné štúdium)
- Conservatoire (Konzervatórium)
- Higher Professional Studies/ Graduate Diploma (Absolventský diplom), Bachelor
- Master, Magister, Doctoral, Engineer, PhD, Extensive study for teaching diploma
- Other: ________________________________
- None of these qualifications
- Don’t know

154. If your partner’s highest educational qualification was obtained in **Hungary**, what is the highest level he/she completed? *Please tick only one box.*

- Certification of the Maturity Examination (Párhuzamos oktatás szakközépiskoláb)
- Upper Secondary General School
  ( Gimnázium)
- Upper Secondary Vocational School (Szakközépiskola)
- Vocational certificate based on NVQL examination (Párhuzamos oktatás szakiskolában, Szakiskola, előkészítő szakiskola, Alapfokú iskolai végzettségre épülő szakképzés, évfolyamra épülő szakképzés)
- Vocational certificate based on NVQL examination (Szakképző évfolyamok középiskola utolsó évfolyamára vagy érettségire épülő nem felsőfokú OKJ szakmákban)
- Tertiary Vocational Program (Felsőfokú szakképzés)
- Bachelor’s Degree (Alapképzés), Master’s Degree (Osztatlan képzés/ Master), University Diploma (Egyetemi szintű alapképzés), College Diploma (Főiskolai szintű alapképzések), Certificate in Specialisation (Szakirányú továbbképzés), Doctorate/PhD
- Other: _____________________________
- None of these qualifications
- Don’t know

155. If your partner’s highest educational qualification was obtained elsewhere, what is the highest level he/she completed?

Country: _______________________________________________________________

Type of school/institution: _______________________________________________________________

Educational programme: _______________________________________________________________

Degree/qualification: _______________________________________________________________

- Don’t know

**SECTION G SOCIOECONOMIC CIRCUMSTANCES**

*This section is about you and your partner’s socioeconomic circumstances, such as your employment and financial situation. We first ask about your employment.*

156. Are you currently a full-time student?

- Yes  No

157. Are you currently employed?

- Yes  No

158. If you are not currently employed, have you ever worked before?

- Yes  No ; *please go to question 167*

159. If you have worked before but you are not working right now, how long ago have you stopped working?

 years and  months ago. Or if less than a month ago:  weeks ago.

160. Are you currently on maternity leave or sick leave?

- Yes  No

*Please answer the following questions about your current job, or about your previous job if you are not working at the moment.*

161. Did/do you work as an employee or are/were you self-employed?

- Employee
- Self-employed with employees (*go to question 163*)
- Self-employed/freelance without employees (*go to question 164*)
- Student in Training (apprentice)

162. How many people work/worked for your employer at the place where you work/worked?

- Work alone
- 1 - 5 people
- 6 - 24 people
- 25 – 49
- 50-499
- 500 or more

163. If you are self-employed, how many people do (did) you employ?

- 1-24  25 or more

164. Do (did) you supervise any other employees? (A supervisor or foreman is responsible for overseeing the work of other employees on a day-to-day basis)

- Yes  No

165. How many hours did or do you work in a typical week?

  hours a week

166. Which of these best describes the sort of work you do/did?

- Modern professional occupations such as: teacher – nurse - physiotherapist – social worker – welfare officer – artist– musician – police officer (sergeant or above) – software designer
- Clerical and intermediate occupations such as: secretary – personal assistant – clerical worker – office clerk – call centre - agent – nursing auxiliary – nursery nurse
- Senior managers or administrators (usually responsible for planning, organising and co-ordinating work, and for finance) such as: finance manager – chief executive
- Technical and craft occupations such as: motor mechanic - fitter – inspector – plumber – printer – tool maker – electrician – gardener – train driver
- Semi-routine manual and service occupations such as: postal worker – machine operative – security guard – caretaker - farm worker – catering assistant – receptionist – sales - assistant
- Routine manual and service occupations such as: HGV driver – van driver – cleaner – porter – packer – sewing machinist – messenger – labourer – waiter/waitress – bar staff
- Middle or junior managers such as: office manager – retail manager – bank manager – restaurant manager – warehouse manager – publican
- Traditional professional occupations such as: accountant - solicitor – medical practitioner – scientist – civil/mechanical engineer
- Other: ________________________________________________________________

*Now we would like to ask about your partner’s employment.*

167. Is your partner currently a full-time student?

- Yes  No

168. Is your partner currently employed?

- Yes  No

169. If your partner is not currently employed, has your partner ever worked before?

- Yes  No ; *please go to question 178*  Don’t know; *please go to question 178*

170. If your partner has worked before but is not working right now, how long ago has your partner stopped working?

 years and  months ago. Or if less than a month ago:  weeks ago.

 Don’t know

171. Is your partner currently on sick leave?

- Yes  No
- Don’t know

*Please answer the following questions about your partner’s current job, or about your partner’s previous job if your partner is not working at the moment.*

172. Did/does your partner work as an employee or is/was your partner self-employed?

- Employee
- Self-employed with employees
  *(go to question 174)*
- Self-employed/freelance without employees *(go to question 175)*
- Student in Training (apprentice)
- Don’t know

173. How many people work/worked for the employer at the place where your partner works/worked?

- Work alone
- 1 - 5 people
- 6 - 24 people
- 25 – 49
- 50-499
- 500 or more
- Don’t know

174. If your partner is self-employed, how many people does (did) your partner employ?

- 1-24  25 or more
- Don’t know

175. Does (did) your partner supervise any other employees? (A supervisor or foreman is responsible for overseeing the work of other employees on a day-to-day basis)

- Yes  No
- Don’t know

176. How many hours did or does your partner work in a typical week?

  hours a week  Don’t know

177. Which of these best describes the sort of work your partner did or does?

- Modern professional occupations such as: teacher – nurse - physiotherapist – social worker – welfare officer – artist– musician – police officer (sergeant or above) – software designer
- Clerical and intermediate occupations such as: secretary – personal assistant – clerical worker – office clerk – call centre - agent – nursing auxiliary – nursery nurse
- Senior managers or administrators (usually responsible for planning, organising and co-ordinating work, and for finance) such as: finance manager – chief executive
- Technical and craft occupations such as: motor mechanic - fitter – inspector – plumber – printer – tool maker – electrician – gardener – train driver
- Semi-routine manual and service occupations such as: postal worker – machine operative – security guard – caretaker - farm worker – catering assistant – receptionist – sales - assistant
- Routine manual and service occupations such as: HGV driver – van driver – cleaner – porter – packer – sewing machinist – messenger – labourer – waiter/waitress – bar staff
- Middle or junior managers such as: office manager – retail manager – bank manager – restaurant manager – warehouse manager – publican
- Traditional professional occupations such as: accountant - solicitor – medical practitioner – scientist – civil/mechanical engineer
- Other: ________________________________________________________________

**THIS SECTION OF THE QUESTIONNAIRE IS SELF-COMPLETED. PLEASE HAND THE PAPER TO PARTICIPANT**

*The next few questions are about your financial situation.*

178. Do you (and your partner) receive any of these benefits/ credits/ allowances?

- Child Benefit
- Child Tax Credit
- Childcare Grant
- Healthy Start
- Maternity Allowance
- Working Tax Credit
- Income Support
- Disability Living Allowance
- Income-based Jobseekers’ allowance
- Income-related Employment and Support allowance
- Housing benefit/ rent rebate/ council tax benefit
- Local Housing Allowance
- Incapacity Benefit
- Pension Credit
- Carer’s Credit
- Universal Credit
- Other: ____________________________________________
- None
- Don’t know
- Do not wish to answer

179-182. This table shows income in weekly, monthly and annual amounts. Which of the amounts on this list represents the **total income of your household**; income from all jobs, (full and part time), all tax credits, all benefits and all other sources and earnings after tax when all income is added together.

*Please tick only one box.*


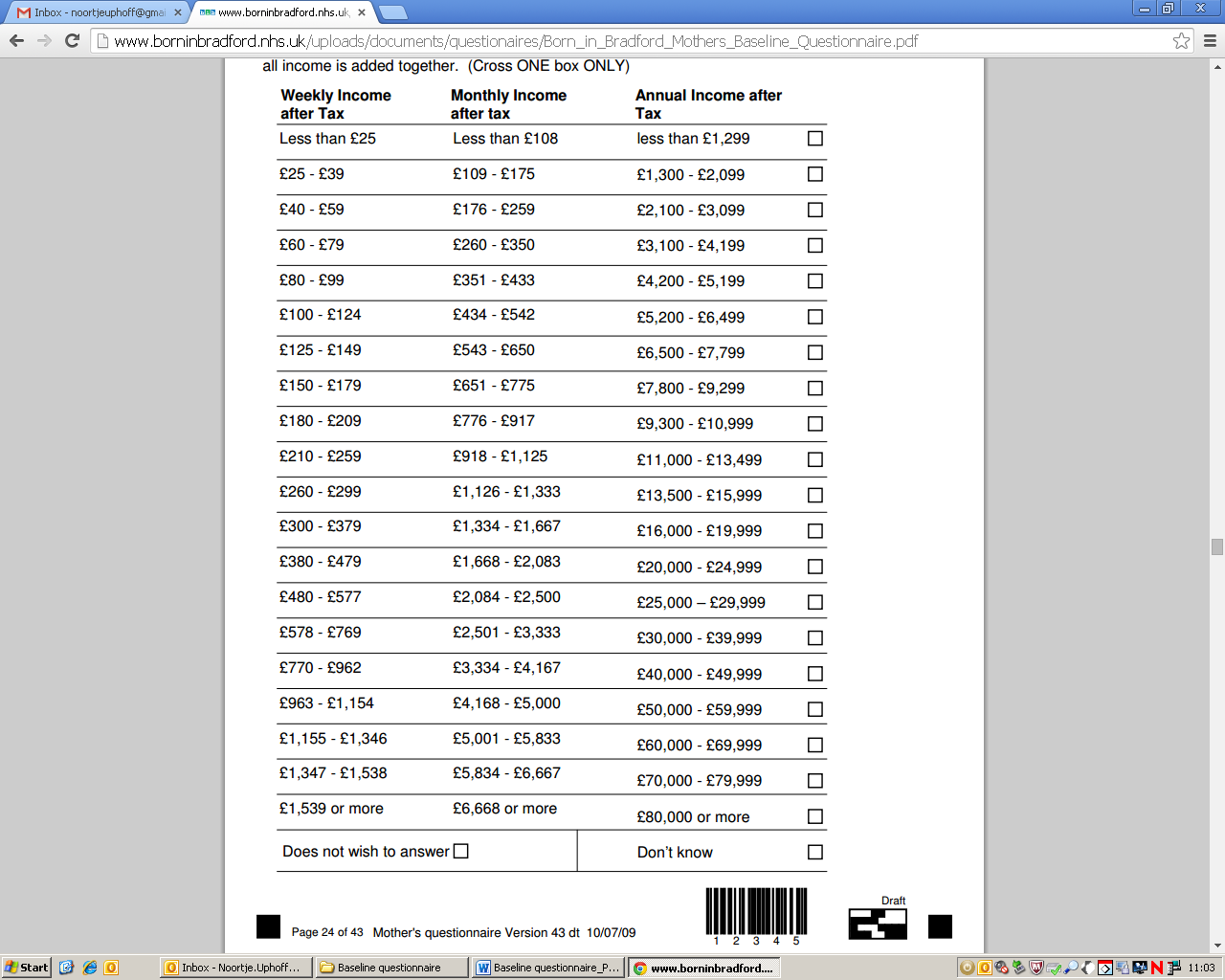


183. Who manages the finances in the household? Tick the box for everyone who manages the household budget, makes decisions about large purchases, pays bills, applies for benefits/tax credit/loans and so on.

- Me
- My partner
- My mother
- My father
- Mother in law
- Father in law
- Someone else: ___________________________
- Don’t know
- Do not wish to answer

184. This question asks about financial resources of household. Do you (and your partner) have:

|  | **Yes** | **No, I would like to but cannot afford this at the moment** | **No, I do not want or need this at the moment** | **No, other reason** | **Do not wish to answer** |
| --- | --- | --- | --- | --- | --- |
| Drinks or a meal with friends or family who don’t live with you at least once a month |  |  |  |  |  |
| Money to make regular savings of £20 a month or more for rainy days or retirement |  |  |  |  |  |
| Money to replace any worn out furniture |  |  |  |  |  |
| Money to replace or repair major electrical goods |  |  |  |  |  |
| A small amount of money to spend on yourself each week |  |  |  |  |  |
| In winter are you able to keep your home warm enough |  |  |  |  |  |
| Fresh fruit and vegetables on a daily basis |  |  |  |  |  |

*These next questions are about food and money during this pregnancy. Please read each statement below and tell us whether the statement was OFTEN, SOMETIMES, or NEVER true for you during this pregnancy.*

185. The food that I bought just didn't last, and I didn't have money to get more.

- Often true
- Sometimes true
- Never true

 Do not wish to answer

186. I couldn't afford to eat balanced meals.

- Often true
- Sometimes true
- Never true

 Do not wish to answer

187. Did you ever cut the size of your meals, eat less or skip meals because there wasn't enough money for food?

- Yes
- No

 Do not wish to answer

188. If yes, how often did this happen?

- Every week
- Not every week but at least once a month
- Less than once a month but a few times
- Don’t know

189. How well would you say you (and your partner) are managing financially these days?
Would you say you are:

- Living comfortably
- Doing alright
- Just about getting by
- Finding it quite difficult
- Finding it very difficult

- Don’t know
- Do not wish to answer

190. Compared to a year ago, how would you say you (and your partner) are doing financially now?

- Better off
- About the same
- Worse off
- Don’t know
- Do not wish to answer

191-192. In the past year have you or you and your partner taken a loan or credit with a short term loan company (e.g. payday loan, BrightHouse, Wonga) to pay for items such as clothing, furniture, phone?

- No
- Yes – 1 time
- Yes – 2 or more times
- Don’t know
- Do not wish to answer

193-194. In the past year have family, friends or relatives lent you or you and your partner money
to pay for items such as clothing, furniture, phone?

- No
- Yes – 1 time
- Yes – 2 or more times
- Don’t know
- Do not wish to answer

195. Sometimes people are not able to pay every bill when it is due. Are you up to date with all bills?

- Yes
- No
- Don’t know
- Do not wish to answer

196. If you are not up to date with some bills, which ones are you behind with?

- Electricity, Gas or other household
  fuel bills
- Council tax
- Insurance Policies
- Mobile or home telephone, internet or television
- Water rates
- Credit cards (bank or store credit cards)
- Short term loans/credit (e.g. payday loan, brighthouse)
- Other bills
- Don’t know
- Do not wish to answer

*Think of this ladder as showing where people stand* ***in your neighbourhood****. By your neighbourhood, I mean within about a mile or 20 minute walk of your home.*

*At the top of the ladder are the people who are the best off – those who have the most money, the best education, and the most respected jobs. At the bottom are the people who are the worst off – who have the least money, least education, and the least respected job or no job.*

*The higher up you are on this ladder, the closer you are to the people at the top; the lower you are, the closer you are to the people at the bottom.*

197. Where would you place yourself on this ladder? Please tick the box next to the rung where you think you stand at this time of your life relative to other people **in your neighbourhood**.


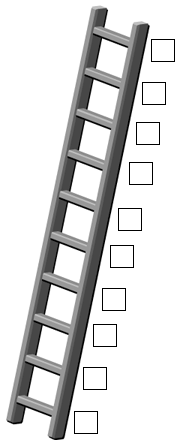


 Do not wish to answer

You have just ranked yourself on the social ladder compared to other people in your neighbourhood. Could you now do the same, but with the ladder representing all people **in England**?

The higher up you are on this ladder, the closer you are to the people at the top; the lower you are, the closer you are to the people at the bottom.

198. Where would you place yourself on this ladder? Please tick the box next to the rung where you think you stand at this time of your life relative to other people **in England**.


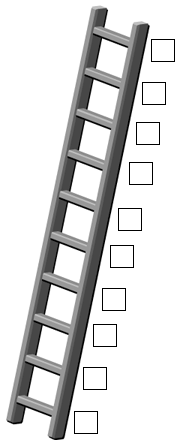


 Do not wish to answer

**SECTION H SOCIAL CIRCUMSTANCES**

*This section is about your relationships with people in your life, social support and activities
 you take part in in your community.*

*If you don’t have a partner, you can skip question H1 and H2 and go to question H3.*

199. My partner doesn’t seem to listen to me

- Strongly agree  Do not wish to answer
- Agree
- Neither agree nor disagree
- Disagree
- Strongly disagree

200. I wish there was more warmth and affection between us

- Strongly agree  Do not wish to answer
- Agree
- Neither agree nor disagree
- Disagree
- Strongly disagree

201. I feel closely attached to my family

- Strongly agree  Do not wish to answer
- Agree
- Neither agree nor disagree
- Disagree
- Strongly disagree

202. My family takes notice of my opinions

- Strongly agree  Do not wish to answer
- Agree
- Neither agree nor disagree
- Disagree
- Strongly disagree

203. Sometimes I feel excluded in my own family

- Strongly agree  Do not wish to answer
- Agree
- Neither agree nor disagree
- Disagree
- Strongly disagree

204. How often do you feel that you, personally, have been discriminated against
because of your ethnicity, race, or religion?

- Never  Do not wish to answer
- Rarely  Don’t know
- Sometimes
- Often

**THIS SECTION IS TO BE COMPLETED BY THE INTERVIEWER. PLEASE HAND THE PAPER BACK TO THE INTERVIEWER.**

205. How many relatives, other than the children that live at home, do you see once a week or more often?

*Include adult relatives living in the home. Please tick only one box.*

0 1 2 3 4 5 6 7 8 9 10 or more

          

 Don’t know

206. In general, would you like to see your relatives:

- More often
- Less often
- It is about right

 Do not wish to answer

207. How many people can you count on in times of need?

0 1 2 3 4 5 6 7 8 9 10 or more

          

 Don’t know

208. *If you don’t have children yet please skip this question and go to H11.*
How many people would be able to take care of your child(ren) for several hours if needed? (including your partner)

0 1 2 3 4 5 6 7 8 9 10 or more

          

 Don’t know

209. How many of these people are from your neighbourhood?

- All
- Most
- Some
- None

210. Are there adults (including your partner) with whom you have regular talks?

- Yes
- No

211. Generally speaking, would you say that most people can be trusted or you can’t be too careful in dealing with people?

- 1 Can be trusted  Don’t know
- 2
- 3
- 4
- 5 Can’t be too careful

212. Do you regularly join in the activities of any organisations or clubs?

- Yes
- No

213. If yes, do you participate in any of these organisations?

- Voluntary community work (such as
  food bank)
- Environmental group
- Political party
- Trade union
- Parent-teacher association
- Tenants group
- Attending a religious group, church or mosque
- Youth group
- Women’s institute
- Social club
- Sports club
- Other: _________________________________

214. Do you know where to find your nearest Children’s centre or community centre?

- Yes
- No

*If you don’t have children yet please skip the next question.*

215. If you have children already, do they regularly attend any of these?

- Nursery
- Playgroup/ toddler group
- Children’s centre activities
- School trips
- None of these

216. We are interested in learning about social networks within the Better Start area.

Could you name another pregnant woman or a woman with children under the age of five?

- No
- Yes ; Name: ______________________________________ Age: 

**SECTION I HEALTH AND WELLBEING**

**THIS SECTION IS SELF-COMPLETED. PLEASE HAND THE PAPER TO THE PARTICIPANT**

*This is the last section of the questionnaire. It is about your health and wellbeing, and includes topics such as health behaviour, mental health and your feelings about the baby.*

233. How would you describe your health generally?

- Excellent
- Very good
- Good
- Fair
- Poor

- Don’t know
- Do not wish to answer

234. If you have a partner at the moment, how would you describe your partner’s health generally?

- Excellent
- Very good
- Good
- Fair
- Poor
- Don’t know
- Do not wish to answer

235. How would you describe the health of your teeth and mouth?

- Excellent
- Very good
- Good
- Fair
- Poor
- Don’t know
- Do not wish to answer

236-237. Did you ever smoke cigarettes or use tobacco (including shisha/hookah, chewing tobacco
and snuff but not e-cigarettes) before you were pregnant?

- Yes, for more than 1 year  Do not wish to answer
- Yes, for 1 year or less
- No

238. If yes, how often did you use tobacco products before you were pregnant?

- less than 1 a day
- 1-5 a day
- 6-10 a day
- 11-20 a day
- 20 a day or more
- Do not wish to answer

239. Since finding out you were pregnant, how often did you or do you use tobacco products?

- None
- Less than one a day  Do not wish to answer
- 1-5 a day
- 6-10 a day
- 11-20 a day
- 20 a day or more

240. Does anybody else smoke in your house (excluding e-cigarettes)?

- Yes
- No
- Do not wish to answer

241. Have you ever used any non-prescription drugs (like cannabis/marijuana or ecstasy) during this pregnancy or in the three months before this pregnancy?

- Yes
- No
- Don’t remember
- Do not wish to answer

242. Did you drink any alcohol during the three months before this pregnancy?

- Yes, once per week or more
- Yes, but less than once a week
- No
- Don’t remember
- Don’t want to answer

243. Do you drink any alcohol during this pregnancy?

- Yes, once per week or more  Do not wish to answer
- Yes, but less than once a week
- No

244-246. If you drink alcohol during this pregnancy, how often do you consume five or more units of alcohol on one occasion?

- Every day
- Nearly every day
- 1 to 4 times a week
- 1 to 3 times a month
- Rarely; less than once a month
- Never
- Don’t know
- Do not wish to answer

247. Who usually does the food shopping in the household?

- Me
- My partner
- Some days me, some days my partner
- Me and my partner together
- My parents or my partner’s parents
- Other: _________________________

248. Who usually does the cooking in the household?

- Me
- My partner
- Some days me, some days my partner
- Me and my partner together
- My parents or my partner’s parents
- Other: _________________________

*Next we would like to ask some questions about your mental health and wellbeing.*

249. Over the last 2 weeks, on how many days have you been bothered by any of the following problems?

|  | **Not at all** | **Several days** | **More than half the days** | **Nearly every day** | **Do not wish to answer** |
| --- | --- | --- | --- | --- | --- |
| 1. Little interest or pleasure in doing things |  |  |  |  |  |
| 2. Feeling down, depressed, or hopeless |  |  |  |  |  |
| 3. Trouble falling or staying asleep, or sleeping too much |  |  |  |  |  |
| 4. Feeling tired or having little energy |  |  |  |  |  |
| 5. Poor appetite or overeating |  |  |  |  |  |
| 6. Feeling bad about yourself — or that you are a failure or have let yourself or your family down |  |  |  |  |  |
| 7. Trouble concentrating on things, such as reading the  newspaper or watching television |  |  |  |  |  |
| 8. Moving or speaking so slowly that other people could have noticed? Or the opposite — being so fidgety or restless that you have been moving around a lot more than usual? |  |  |  |  |  |
| 9. Thoughts that you would be better off dead or of hurting yourself in someway |  |  |  |  |  |

250. If you checked off any problems, how difficult have these problems made it for you to do your work, take care of things at home, or get along with other people?

- Not difficult at all
- Somewhat difficult
- Very difficult
- Extremely difficult

*Below are some questions that ask about your circumstances and feelings around the time you became pregnant with this baby.*

251. In terms of becoming a mother (first time or again), I feel that my pregnancy happened at the…
*(please tick the statement which most applies to you)*

- right time
- ok, but not quite right time
- wrong time

252. Just before I became pregnant…
*(please tick the statement which most applies to you)*

- I intended to get pregnant
- my intentions kept changing
- I did not intend to get pregnant

253. Before I became pregnant…
*(please tick the statement which most applies to you)*

- the father of the baby and I had agreed that we would like me to be pregnant
- the father of the baby and I had discussed having children together, but hadn’t agreed

for me to get pregnant

- we never discussed having children together

*These questions are about your thoughts and feelings about the developing baby.*

*Please tick one box only in answer to each question.*

254. Over the past two weeks I have thought about,
or been preoccupied with the baby inside me:

- Almost all the time
- Very frequently
- Frequently
- Occasionally
- Not at all
- Do not wish to answer

255. Over the past two weeks when I have spoken about, or thought about the baby inside me I got emotional feelings which were:

- Very weak or non-existent
- Fairly weak
- In between strong and weak
- Fairly strong
- Very strong
- Do not wish to answer

256. Over the past two weeks my feelings about the baby inside me have been:

- Very positive
- Mainly positive
- Mixed positive and negative
- Mainly negative
- Very negative
- Do not wish to answer

257. Over the past two weeks I have had the desire to read about or get information about the developing baby. This desire is:

- Very weak or non-existent
- Fairly weak
- Neither strong nor weak
- Moderately strong
- Very strong
- Do not wish to answer

258. Over the past two weeks I have been trying
to picture in my mind what the developing baby actually looks like in my womb:

- Almost all the time
- Very frequently
- Frequently
- Occasionally
- Not at all
- Do not wish to answer

259. Over the past two weeks I think of the developing baby mostly as:

- A real little person with special characteristics
- A baby like any other baby
- A human being
- A living thing
- A thing not yet really alive
- Do not wish to answer

260. Over the past two weeks I have felt that the baby inside me is dependent on me for its well-being:

- Totally
- A great deal
- Moderately
- Slightly

- Not at all
- Do not wish to answer

261. Over the past two weeks I have found myself talking to my baby when I am alone

- Not at all
- Occasionally
- Frequently

- Very frequently
- Almost all the time I am alone
- Do not wish to answer

262. Over the past two weeks when I think about
(or talk to) my baby inside me, my thoughts:

- Are always tender and loving
- Are mostly tender and loving
- Are a mixture of both tenderness and irritation
- Contain a fair bit of irritation
- Contain a lot of irritation
- Do not wish to answer

263. The picture in my mind of what the baby at this stage actually looks like inside the womb is:

- Very clear
- Fairly clear
- Fairly vague
- Very vague
- I have no idea at all
- Do not wish to answer

264. Over the past two weeks when I think about
the baby inside me I get feelings which are:

- Very sad
- Moderately sad
- A mixture of happiness and sadness
- Moderately happy
- Very happy
- Do not wish to answer

265. Over the past two weeks I have felt:

- Very emotionally distant from my baby
- Moderately emotionally distant from my baby
- Not particularly emotionally close to my baby
- Moderately close emotionally to my baby
- Very close emotionally to my baby
- Do not wish to answer

266. Over the past two weeks I have taken care with
what I eat to make sure the baby gets a good diet:

- Not at all
- Once or twice when I ate
- Occasionally when I ate
- Quite often when I ate
- Every time I ate
- Do not wish to answer

267. When I first see my baby after the birth
I expect I will feel:

- Intense affection
- Mostly affection
- Dislike about one or two aspects of the baby
- Dislike about quite a few aspects of the baby
- Mostly dislike
- Do not wish to answer

268. When my baby is born I would like to hold
the baby:

- Immediately
- After it has been wrapped in a blanket
- After it has been washed
- After a few hours for things to settle down
- The next day
- Do not wish to answer

269. Over the past two weeks I have had dreams
about the pregnancy or baby:

- Not at all
- Occasionally
- Frequently
- Very frequently
- Almost every night
- Do not wish to answer

270. Over the past two weeks I have found myself feeling, or
rubbing with my hand, the outside of my stomach where the baby is:

- A lot of times each day
- At least once per day
- Occasionally
- Once only
- Not at all
- Do not wish to answer

*Now we would like to ask about your plans for feeding the baby. To what extent do you agree with the following statements?*

271. I am planning to only formula feed my baby
(I will not breastfeed at all)

- Very much agree
- Somewhat agree
- Unsure
- Somewhat disagree
- Very much disagree
- Don’t know

272. I am planning to at least give breastfeeding a try

- Very much agree
- Somewhat agree
- Unsure
- Somewhat disagree
- Very much disagree
- Don’t know

273. When my baby is 1 month old, I will be
breastfeeding without using any formula or
other milk

- Very much agree
- Somewhat agree
- Unsure
- Somewhat disagree
- Very much disagree
- Don’t know

274. When my baby is 3 month old, I will be breastfeeding without using any formula or
other milk

- Very much agree
- Somewhat agree
- Unsure
- Somewhat disagree
- Very much disagree
- Don’t know

275. When my baby is 6 month old, I will be
breastfeeding without using any formula or
other milk

- Very much agree
- Somewhat agree
- Unsure
- Somewhat disagree
- Very much disagree
- Don’t know

*The following questions ask about arguments between you and your partner. If you do not have a partner at the moment you may skip these questions and go to question I47.*

276. Many couples argue from time to time.

Roughly how often do you and your partner argue?

- Most days
- At least once a week
- Less than once a week
- Hardly ever
- Never

277. When you and your partner argue, do you shout
or yell at each other?

- Never
- Not very often
- Sometimes
- Often
- Almost always/ always

278. When you and your partner argue, do you
throw something at each other?

- Never
- Not very often
- Sometimes
- Often
- Almost always/ always

279. When you and your partner argue,
do you push, hit or slap each other?

- Never
- Not very often
- Sometimes
- Often
- Almost always/ always

 Do not wish to answer these questions

*The next set of questions is about your diet.*

280. How often do you eat these foods:

|  | 6+ times per day | 4-5 times per day | 2-3 times per day | Once per day | 5-6 times per week | 2-4 times per week | Once per week | 1-3 times per month | Less than once a month |
| --- | --- | --- | --- | --- | --- | --- | --- | --- | --- |
| Breakfast Cereal |  |  |  |  |  |  |  |  |  |
| Fresh fruit |  |  |  |  |  |  |  |  |  |
| Cooked green vegetables (fresh or frozen) |  |  |  |  |  |  |  |  |  |
| Cooked root vegetables (fresh or frozen) |  |  |  |  |  |  |  |  |  |
| Raw vegetables or salad (including tomatoes) |  |  |  |  |  |  |  |  |  |
| Chips |  |  |  |  |  |  |  |  |  |
| Potatoes, pasta, rice |  |  |  |  |  |  |  |  |  |
| Meat |  |  |  |  |  |  |  |  |  |
| Poultry |  |  |  |  |  |  |  |  |  |
| White Fish |  |  |  |  |  |  |  |  |  |
| Oil rich fish |  |  |  |  |  |  |  |  |  |
| Cheese |  |  |  |  |  |  |  |  |  |
| Beans or pulses |  |  |  |  |  |  |  |  |  |
| Sweets, chocolates |  |  |  |  |  |  |  |  |  |
| Ice cream |  |  |  |  |  |  |  |  |  |
| Crisps, savoury snacks |  |  |  |  |  |  |  |  |  |
| Fruit juice (NOT squash) |  |  |  |  |  |  |  |  |  |
| Soft/fizzy drinks |  |  |  |  |  |  |  |  |  |
| Cakes, scones, sweet pies or pastries |  |  |  |  |  |  |  |  |  |
| Biscuits |  |  |  |  |  |  |  |  |  |

*This is the last set of questions of this questionnaire. It is about your mental wellbeing.*

281. Over the last 2 weeks, on how many days have you been bothered by any of the following problems?

|  | **Not at all** | **Several days** | **More than half the days** | **Nearly every day** | **Do not wish to answer** |
| --- | --- | --- | --- | --- | --- |
| 1. Feeling nervous, anxious or on an edge? |  |  |  |  |  |
| 2. Not being able to stop or control worrying? |  |  |  |  |  |
| 3. Worrying too much about different things? |  |  |  |  |  |
| 4. Trouble relaxing? |  |  |  |  |  |
| 5. Being so restless that it is hard to sit still? |  |  |  |  |  |
| 6. Becoming easily annoyed or irritable? |  |  |  |  |  |
| 7. Feeling afraid as if something awful might happen? |  |  |  |  |  |

 Do not wish to answer these questions

This is the end of the questionnaire. If you have any questions about our study, please ask the researcher. Thank you very much for your help!

**Appendix 3: Shortened Version of the Baseline Questionnaire for Pregnant Women**

**
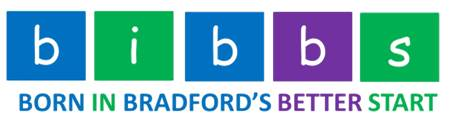
**

**BiBBS questionnaire for pregnant women**

This questionnaire is for pregnant women in the areas Little Horton, Bowling & Barkerend and Bradford Moor. It usually takes about 20 minutes to complete.

This questionnaire is about you and your baby. We are interested to know about your family life and relationships, your house and neighbourhood, the languages you speak, your social and financial circumstances, your health and wellbeing, this pregnancy and your plans for the baby.

We would be grateful if you help us by answering as many of these questions as possible but if there are any questions you do not want to answer that is fine. There are no right or wrong answers.

All the answers you give are confidential. Your name and full address will not appear anywhere on the questionnaire.

We apologise if any questions cause offence – this is not our intention. We are asking everyone the same questions but we realise you may find some questions odd or unusual.

We will start by taking some measurements of your height, weight and arm size.

Thank you for being a part of our BIBBS study!


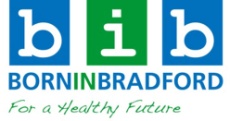

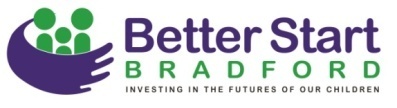

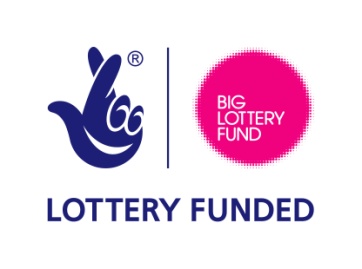


**FRONT SHEET**

**-------------------------------------- TO BE COMPLETED BY RESEARCHER --------------------------------------**

PARTICIPANT INFORMATION

Participant Study ID _______________________________________________

1. Date completing questionnaire   

2. Who is administering the questionnaire? ______________________________

3. Language used for administration  English

 Punjabi

 Urdu

 Other: ____________

4. Interpreter used  No

 Yes, family or friend

 Yes, other

6. Who is present at the interview  Partner

 Family member

 Friend

 Other

 None

8. Gestational age at completion before birth  weeks  Don’t know

after birth  weeks

MEASUREMENTS

9. Who is taking the measurements? ­­­­­­­­­­­________________________________

10. Height   cms  Not able to take

12. Weight   kgs  Not able to take
16. Triceps   cms  Not able to take

**SECTION A BACKGROUND**

*This first section is about your background.*

18. What is your date of birth?

  

Day Month Year

19. What country were you born in?

 England  Northern Ireland  Poland

 Pakistan  Scotland  Czech Republic

 Bangladesh  Wales  Slovakia

 India  Republic of Ireland  Romania

 Hungary

 Other: ________________________

20. What country were your parents born in?

Your mother: Your father:

  England

  Pakistan

  Bangladesh

  India

  Northern Ireland

  Scotland

  Wales

  Republic of Ireland

  Slovakia

  Czech Republic

  Poland

  Romania

  Hungary

  Other: __________________________________________________

  Don’t know

22. If you were not born in the UK, how old were you when you moved to the UK?

 years old  Don’t know

23. What best describes your ethnic group or background?

- White; English/Welsh/Scottish/Northern Irish/British
- White; Irish
- Pakistani
- Indian
- Bangladeshi
- White; Polish
- White; Slovakian
- White; Romanian
- White; Czech
- Other White
- White; Gypsy/Roma or Irish traveller
- Chinese
- African
- Caribbean
- Mixed White and Black Caribbean
- Mixed White and Black African
- Mixed White and Asian
- Any other mixed/multiple ethnic background: ________________________________________
- Any other ethnic background: ________________________________________

24. What is your religion?

- None
- Christian (including Church of England, Catholic, Protestant and all Christian denominations)
- Islam
- Sikhism
- Buddhism
- Hinduism
- Judaism
- Any other religion: ________________________________________

**SECTION B HOUSEHOLD INFORMATION**

*This section is about the people in your household and their relationships. If you are unsure please ask the researcher – he or she can help you with these questions.*

25. How many people live in your household, including yourself?  people

26. How many children do you already have?  children

27. What is your relationship with the baby’s natural father?

- Married to baby’s father
- In a relationship with baby’s father but not married
- Separated or divorced
- Never been in a relationship with baby’s father
- Baby’s father has died

28. Are you living with the baby’s natural father?

- Yes
- No

29. Do you currently have a partner?

- Yes
- No

30. If you have a partner at the moment, what is your partner’s date of birth?

    Don’t know

Day Month Year

31. Which country was the father of your baby born in?

🞏 England 🞏 Northern Ireland 🞏 Slovakia

🞏 Pakistan 🞏 Scotland 🞏 Czech Republic

🞏 Bangladesh 🞏 Channel Islands 🞏 Poland

🞏 India 🞏 Isle of Man 🞏 Philippines

🞏 Republic of Ireland 🞏 Other: _________________________

🞏 Don’t know

32. Are you related to the father of your baby other than by marriage?
*If you are unsure please ask the researcher – he or she can help you with this question.*

🞏 Yes

🞏 No

🞏 Don’t know

🞏 Do not wish to answer

33. If yes, how are you related to the father of your baby?
*If you are unsure please ask the researcher – he or she can help you with this question.*

🞏 First cousin

🞏 First cousin, once removed

🞏 Second cousin

🞏 Other related by blood

🞏 Don’t know

**SECTION C HOUSE**

*This next section is about the house you live in at the moment and your previous home.*

34. What is your postcode?  

35. How long have you lived at your current address?

 years and  months

**SECTION D NEIGHBOURHOOD**

*This next section is about the neighbourhood you live in.*

36. How satisfied or dissatisfied are you with the area you live in?
*By your area, I mean within about a mile or 20 minute walk of your home.*

- Very satisfied
- Fairly satisfied
- Neither satisfied nor dissatisfied
- Fairly dissatisfied
- Very dissatisfied

37. How satisfied or dissatisfied are you with the parks and green spaces in your local area?

*By your area, I mean within about a mile or 20 minute walk of your home.*

- Very satisfied
- Fairly satisfied
- Neither satisfied nor dissatisfied
- Fairly dissatisfied
- Very dissatisfied

38. Thinking about your neighbourhood, to what extent do you agree with this statement:

*Other people think this is a good area.*

- Strongly agree
- Agree
- Neither agree nor disagree
- Disagree
- Strongly disagree

**SECTION E LANGUAGE**

*This next section is about languages you speak, languages in your households and books in your home.*

39. What is your first language?

*Please tick two boxes if you are bilingual (you speak two languages fluently and were taught these languages from a young age).*

- English
- Punjabi
- Urdu (including Hindi)
- Polish
- Slovakian
- Hungarian
- Romanian
- Russian
- Gujarati
- Spanish
- Pashto
- Bengali (including Sylheti, Chatgaya/Chittagonian)
- Arabic
- Other: ______________________

40. Which of these languages are usually spoken at home?

- English
- Punjabi
- Urdu (including Hindi)
- Polish
- Slovakian
- Hungarian
- Romanian
- Russian
- Gujarati
- Spanish
- Pashto
- Bengali (including Sylheti, Chatgaya/Chittagonian)
- Arabic
- Other: ___________________

41. If English is not your first language, how well can you do the following things **in English**:

**Not at all A little bit Some Quite well Very well**

Listening     

Reading     

Writing     

Speaking     

42. If you have preschool children or children in primary school already, how many days in a typical week do you read with them?

- 0
- 1
- 2
- 3
- 4
- 5
- 6
- 7

**SECTION F EDUCATION**

*This next section is about you and your partners’ education. Please skip the questions that don’t apply to you. We first ask about your own education.*

43. Where did you obtain your highest educational qualification?

- England
- Pakistan
- India
- Bangladesh
- Poland
- Slovakia
- Hungary
- Elsewhere

44. If your highest educational qualification was obtained in **England**, what is the highest level you completed? *Please tick only one box.*

- Less than 5 GCSEs (A*-C), CSE or O-Levels
- 5 or more GCSEs (A*-C), SCEs or O-Levels
- GNVQ foundation level
- NVQ1
- GNVQ intermediate
- NVQ 2
- (Young) apprenticeship
- NVQ 3
- Advanced apprenticeship
- GNVQ Advanced
- AS or A level, International Baccalaureate or BTEC
- NVQ 4/5
- Higher National Certificate/ Higher National Diploma, Higher Education Diploma
- Foundation Degree
- Bachelor’s degree 3-5 years
- Master’s degree (taught/research) or Postgraduate qualification, Doctorate/ PhD
- Other: _______________________________
- None of these qualifications
- Don’t know

45. If your highest educational qualification was obtained in **Pakistan**, what is the highest level you completed? *Please tick only one box.*

- Secondary School Certificate/ Matriculation
- Higher Secondary or Intermediate Certificate
- Technical Education Certificate/ Diploma
- Registered Nurse/ General Nursing Diploma
- Primary Teaching Certificate
- Certificate in Training, Paramedic Secondary School Certificate/ Matriculation
- Bachelor Degree Arts/Sciences, Education, Engineering, Medicine/Surgery
- Master Degree/ Master of Education/ Master of Philosophy (MPhil), Doctorate
- Other: ________________________________
- None of these qualifications
- Don’t know

46. If your highest educational qualification was obtained in **India**, what is the highest level you completed? *Please tick only one box.*

- Senior Secondary School Leaving Certificate
- Matriculation Certificate, Senior School Certificate, ITI Certificate
- Diploma in Technical Education, Junior basic teacher’s training, Nursing Diploma/Certificate
- Bachelor’s Degree, Bachelor of Law/ Education
- Master’s Degree, Master of Philosophy (Mphil), Doctor of Philosophy/ Letters
- Other: ________________________________
- None of these qualifications
- Don’t know

47. If your highest educational qualification was obtained in **Bangladesh**, what is the highest level you completed? *Please tick only one box.*

- Secondary School/ Higher Secondary Certificate
- Dakhil/ Alim Certificate
- SSC Vocational/ Trade Certificate
- HSC Business Management
- HSC Vocational
- Diploma in Commerce Certificate
- Diploma in Engineering
- Certificate in Education (CinEd)
- Diploma in Agriculture/Ayurvedic & Unanai Medicine/ Textile/ Health Technology/ Survey/ Ceramics/ Nursing/ Graphic Arts
- Bachelor Degree Pass, Bachelor Degree
- Master’s Degree, PGD and PhD
- Fazil/ Kamil Degree
- Other: ________________________________
- None of these qualifications
- Don’t know

48. If your highest educational qualification was obtained in **Poland**, what is the highest level you completed? *Please tick only one box.*

- General Secondary School Leaving Certificate/ Diploma
- Technikum, Liceum Mature Certificate/Diploma
- Technical Secondary School, Specialised Secondary School
- General/ Supplementary Secondary School
- Ballet School Diploma (technician level), Second level music school Diploma, Circus School Diploma (technician level)
- Basic vocational School
- Post-secondary School Certificate/ Diploma
- Teacher Training, Diploma Social Work College
- 1st Degree Studies
- University Studies, Magister/Lekarz, Postgraduate Certificate
- Master’s Degree, Doctoral Study/PhD
- Other: ________________________________
- None of these qualifications
- Don’t know

49. If your highest educational qualification was obtained in **Slovakia**, what is the highest level you completed? *Please tick only one box.*

- Study of selected subjects (Štúdium jednotlivých predmetov)
- Vocational School (Odborné učilište)
- Secondary Specialised School without maturita (Vysvedčenie o záverečnej skúške, Výučný list)
- Retraining Courses Certificate (Osvedčenie)
- Secondary Specialised School with maturita (Vysvedčenie o maturitnej skúške, Vysvedčenie o maturitnej skúške, Výučný list)
- Gymnasium (gymnázium)
- Follow-up courses (Nadstavbové štúdium)
- Post-secondary Qualification Study (Pomaturitné kvalifikačné štúdium)
- Supplementary Pedagogical Study (Doplňujúce pedagogické štúdium)
- Post-secondary Specialised Study (Pomaturitné špecializačné štúdium)
- Conservatoire (Konzervatórium)
- Higher Professional Studies/ Graduate Diploma (Absolventský diplom), Bachelor
- Master, Magister, Doctoral, Engineer, PhD, Extensive study for teaching diploma
- Other: ________________________________
- None of these qualifications
- Don’t know

50. If your highest educational qualification was obtained in **Hungary**, what is the highest level you completed? *Please tick only one box.*

- Certification of the Maturity Examination (Párhuzamos oktatás szakközépiskoláb)
- Upper Secondary General School
  ( Gimnázium)
- Upper Secondary Vocational School (Szakközépiskola)
- Vocational certificate based on NVQL examination (Párhuzamos oktatás szakiskolában, Szakiskola, előkészítő szakiskola, Alapfokú iskolai végzettségre épülő szakképzés, évfolyamra épülő szakképzés)
- Vocational certificate based on NVQL examination (Szakképző évfolyamok középiskola utolsó évfolyamára vagy érettségire épülő nem felsőfokú OKJ szakmákban)

- Tertiary Vocational Program (Felsőfokú szakképzés)
- Bachelor’s Degree (Alapképzés), Master’s Degree (Osztatlan képzés/ Master), University Diploma (Egyetemi szintű alapképzés), College Diploma (Főiskolai szintű alapképzések), Certificate in Specialisation (Szakirányú továbbképzés), Doctorate/PhD
- Other: _____________________________
- None of these qualifications
- Don’t know

51. If your highest educational qualification was obtained elsewhere, what is the highest level you completed?

Country: _______________________________________________________________

Type of school/institution: _______________________________________________________________

Educational programme: _______________________________________________________________

Degree/qualification: _______________________________________________________________

- Don’t know

*The next few questions are about the education of your partner. If you don’t have a partner please go to question 61.*

52. Where did your partner obtain his/her highest educational qualification?

- England
- Pakistan
- India
- Bangladesh
- Poland
- Slovakia
- Hungary
- Elsewhere
- Don’t know

53. If your partner’s highest educational qualification was obtained in **England**, what is the highest level he/she completed? *Please tick only one box.*

- Less than 5 GCSEs (A*-C), CSE or O-Levels
- 5 or more GCSEs (A*-C), SCEs or O-Levels
- GNVQ foundation level
- NVQ1
- GNVQ intermediate
- NVQ 2
- (Young) apprenticeship
- NVQ 3
- Advanced apprenticeship
- GNVQ Advanced
- AS or A level, International Baccalaureate or BTEC
- NVQ 4/5
- Higher National Certificate/ Higher National Diploma, Higher Education Diploma
- Foundation Degree
- Bachelor’s degree 3-5 years
- Master’s degree (taught/research) or Postgraduate qualification, Doctorate/ PhD
- Other: _______________________________
- None of these qualifications
- Don’t know

54. If your partner’s highest educational qualification was obtained in **Pakistan**, what is the highest level he/she completed? *Please tick only one box.*

- Secondary School Certificate/ Matriculation
- Higher Secondary or Intermediate Certificate
- Technical Education Certificate/ Diploma
- Registered Nurse/ General Nursing Diploma
- Primary Teaching Certificate
- Certificate in Training, Paramedic Secondary School Certificate/ Matriculation
- Bachelor Degree Arts/Sciences, Education, Engineering, Medicine/Surgery
- Master Degree/ Master of Education/ Master of Philosophy (MPhil), Doctorate
- Other: ________________________________
- None of these qualifications
- Don’t know

55. If your partner’s highest educational qualification was obtained in **India**, what is the highest level he/she completed?

*Please tick only one box.*

- Senior Secondary School Leaving Certificate
- Matriculation Certificate, Senior School Certificate, ITI Certificate
- Diploma in Technical Education, Junior basic teacher’s training, Nursing Diploma/Certificate
- Bachelor’s Degree, Bachelor of Law/ Education
- Master’s Degree, Master of Philosophy (Mphil), Doctor of Philosophy/ Letters
- Other: ________________________________
- None of these qualifications
- Don’t know

56. If your partner’s highest educational qualification was obtained in **Bangladesh**, what is the highest level he/she completed? *Please tick only one box.*

- Secondary School/ Higher Secondary Certificate
- Dakhil/ Alim Certificate
- SSC Vocational/ Trade Certificate
- HSC Business Management
- HSC Vocational
- Diploma in Commerce Certificate
- Diploma in Engineering
- Certificate in Education (CinEd)
- Diploma in Agriculture/Ayurvedic & Unanai Medicine/ Textile/ Health Technology/ Survey/ Ceramics/ Nursing/ Graphic Arts
- Bachelor Degree Pass, Bachelor Degree
- Master’s Degree, PGD and PhD
- Fazil/ Kamil Degree
- Other: ________________________________
- None of these qualifications
- Don’t know

57. If your partner’s highest educational qualification was obtained in **Poland**, what is the highest level he/she completed? *Please tick only one box.*

- General Secondary School Leaving Certificate/ Diploma
- Technikum, Liceum Mature Certificate/Diploma
- Technical Secondary School, Specialised Secondary School
- General/ Supplementary Secondary School
- Ballet School Diploma (technician level), Second level music school Diploma, Circus School Diploma (technician level)
- Basic vocational School
- Post-secondary School Certificate/ Diploma
- Teacher Training, Diploma Social Work College
- 1st Degree Studies
- University Studies, Magister/Lekarz, Postgraduate Certificate
- Master’s Degree, Doctoral Study/PhD
- Other: ________________________________
- None of these qualifications
- Don’t know

58. If your partner’s highest educational qualification was obtained in **Slovakia**, what is the highest level he/she completed? *Please tick only one box.*

- Study of selected subjects (Štúdium jednotlivých predmetov)
- Vocational School (Odborné učilište)
- Secondary Specialised School without maturita (Vysvedčenie o záverečnej skúške, Výučný list)
- Retraining Courses Certificate (Osvedčenie)
- Secondary Specialised School with maturita (Vysvedčenie o maturitnej skúške, Vysvedčenie o maturitnej skúške, Výučný list)
- Gymnasium (gymnázium)
- Follow-up courses (Nadstavbové štúdium)
- Post-secondary Qualification Study (Pomaturitné kvalifikačné štúdium)
- Supplementary Pedagogical Study (Doplňujúce pedagogické štúdium)
- Post-secondary Specialised Study (Pomaturitné špecializačné štúdium)
- Conservatoire (Konzervatórium)
- Higher Professional Studies/ Graduate Diploma (Absolventský diplom), Bachelor
- Master, Magister, Doctoral, Engineer, PhD, Extensive study for teaching diploma
- Other: ________________________________
- None of these qualifications
- Don’t know

59. If your partner’s highest educational qualification was obtained in **Hungary**, what is the highest level he/she completed? *Please tick only one box.*

- Certification of the Maturity Examination (Párhuzamos oktatás szakközépiskoláb)
- Upper Secondary General School
  ( Gimnázium)
- Upper Secondary Vocational School (Szakközépiskola)
- Vocational certificate based on NVQL examination (Párhuzamos oktatás szakiskolában, Szakiskola, előkészítő szakiskola, Alapfokú iskolai végzettségre épülő szakképzés, évfolyamra épülő szakképzés)
- Vocational certificate based on NVQL examination (Szakképző évfolyamok középiskola utolsó évfolyamára vagy érettségire épülő nem felsőfokú OKJ szakmákban)

- Tertiary Vocational Program (Felsőfokú szakképzés)
- Bachelor’s Degree (Alapképzés), Master’s Degree (Osztatlan képzés/ Master), University Diploma (Egyetemi szintű alapképzés), College Diploma (Főiskolai szintű alapképzések), Certificate in Specialisation (Szakirányú továbbképzés), Doctorate/PhD
- Other: _____________________________
- None of these qualifications
- Don’t know

60. If your partner’s highest educational qualification was obtained elsewhere, what is the highest level he/she completed?

Country: _______________________________________________________________

Type of school/institution: _______________________________________________________________

Educational programme: _______________________________________________________________

Degree/qualification: _______________________________________________________________

- Don’t know

**SECTION G SOCIOECONOMIC CIRCUMSTANCES**

*This section is about you and your partner’s socioeconomic circumstances, such as your employment and financial situation.*

61. Are you currently employed?

- Yes  No

62. If you have a partner, is your partner currently employed?

- Yes  No

**THIS SECTION OF THE QUESTIONNAIRE IS SELF-COMPLETED. PLEASE HAND THE PAPER TO PARTICIPANT.**

*The next few questions are about your financial situation*

63. How well would you say you (and your partner) are managing financially these days?
Would you say you are:

- Living comfortably
- Doing alright
- Just about getting by
- Finding it quite difficult
- Finding it very difficult

- Don’t know
- Do not wish to answer

**SECTION H SOCIAL CIRCUMSTANCES**

*This section is about your relationships with people in your life, social support and activities
 you take part in in your community.*

**THIS SECTION IS TO BE COMPLETED BY THE INTERVIEWER. PLEASE HAND THE PAPER BACK TO THE INTERVIEWER**

64. How many relatives, other than the children that live at home, do you see once a week or more often?

*Include adult relatives living in the home. Please tick only one box.*

0 1 2 3 4 5 6 7 8 9 10 or more

          

 Don’t know

65. How many people can you count on in times of need?

0 1 2 3 4 5 6 7 8 9 10 or more

          

 Don’t know

66. Do you regularly join in the activities of any organisations or clubs?

- Yes
- No

67. Do you know where to find your nearest Children’s centre or community centre?

- Yes
- No

*If you don’t have children yet please skip the next question.*

68. If you have children already, do they regularly attend any of these?

- Nursery
- Playgroup/ toddler group
- Children’s centre activities
- School trips
- None of these

**SECTION I HEALTH AND WELLBEING**

**THIS SECTION IS SELF-COMPLETED. PLEASE HAND THE PAPER TO THE PARTICIPANT**

*This is the last section of the questionnaire. It is about your health and wellbeing, and includes topics such as health behaviour, mental health and your feelings about the baby.*

69. How would you describe your health generally?

- Excellent
- Very good
- Good
- Fair
- Poor

- Don’t know
- Do not wish to answer

70. Since finding out you were pregnant, how often did you or do you use tobacco products?

- None
- Less than one a day  Do not wish to answer
- 1-5 a day
- 6-10 a day
- 11-20 a day
- 20 a day or more

71. If you drink alcohol during this pregnancy, how often do you consume five or more units of alcohol on one occasion?

- Every day
- Nearly every day
- 1 to 4 times a week
- 1 to 3 times a month
- Rarely; less than once a month
- Never
- Don’t know
- Do not wish to answer

*Next we would like to ask some questions about your mental health and wellbeing.*

72. Over the last 2 weeks, on how many days have you been bothered by any of the following problems?

|  | **Not at all** | **Several days** | **More than half the days** | **Nearly every day** | **Do not wish to answer** |
| --- | --- | --- | --- | --- | --- |
| 1. Little interest or pleasure in doing things |  |  |  |  |  |
| 2. Feeling down, depressed, or hopeless |  |  |  |  |  |
| 3. Trouble falling or staying asleep, or sleeping too much |  |  |  |  |  |
| 4. Feeling tired or having little energy |  |  |  |  |  |
| 5. Poor appetite or overeating |  |  |  |  |  |
| 6. Feeling bad about yourself — or that you are a failure or have let yourself or your family down |  |  |  |  |  |
| 7. Trouble concentrating on things, such as reading the  newspaper or watching television |  |  |  |  |  |
| 8. Moving or speaking so slowly that other people could have noticed? Or the opposite — being so fidgety or restless that you have been moving around a lot more than usual? |  |  |  |  |  |
| 9. Thoughts that you would be better off dead or of hurting yourself in someway |  |  |  |  |  |

73. If you checked off any problems, how difficult have these problems made it for you to do your work, take care of things at home, or get along with other people?

- Not difficult at all
- Somewhat difficult
- Very difficult
- Extremely difficult

*These questions are about your thoughts and feelings about the developing baby.*

*If you have already had your baby please skip to question 77.*

*Please tick one box only in answer to each question.*

74. Over the past two weeks when I have spoken about, or thought about the baby inside me I got emotional feelings which were:

- Very weak or non-existent
- Fairly weak
- In between strong and weak
- Fairly strong
- Very strong
- Do not wish to answer

75. Over the past two weeks I have found myself feeling, or rubbing with my hand, the outside of my stomach where the baby is:

- A lot of times each day
- At least once per day
- Occasionally
- Once only
- Not at all
- Do not wish to answer

*Now we would like to ask about your plans for feeding the baby. To what extent do you agree with the following statement?*

76. I am planning to at least give breastfeeding a try

- Very much agree
- Somewhat agree
- Unsure
- Somewhat disagree
- Very much disagree
- Don’t know

77. Many couples argue from time to time.

Roughly how often do you and your partner argue?

- Most days
- At least once a week
- Less than once a week
- Hardly ever
- Never

This is the end of the questionnaire.
If you have any questions about our study, please ask the researcher.

Thank you very much for your help!

**Appendix 4: Baseline Questionnaire for Partners**

**
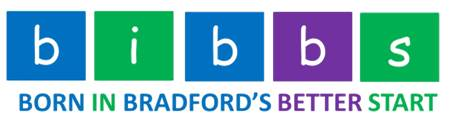
**

**BiBBS questionnaire for partners**

This questionnaire is for partners of pregnant women in the areas Little Horton, Bowling & Barkerend and Bradford Moor. It will take about 20 minutes to complete.

We would like to ask about your circumstances, your family life and environment, your health, and your involvement with this pregnancy. This will give us information on things that may be important to the health and wellbeing of baby’s and young families. We will use this to improve health services for families in the Better Start areas.

We would be grateful if you could help us by answering as many of these questions as possible but if there are any questions you do not want to answer that is fine. There are no right or wrong answers.

All the answers you give are confidential. Your name and full address will not appear anywhere on the questionnaire.

We apologise if any questions cause offence – this is not our intention. We are asking everyone the same questions but we realise you may find some questions odd or unusual.

Thank you for being a part of our BIBBS study!

**
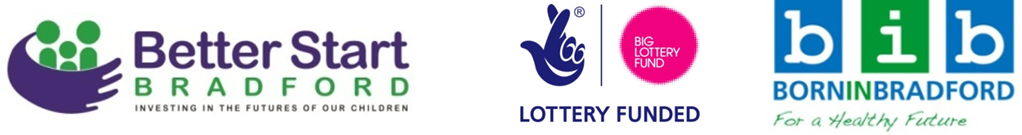
**

**FRONT SHEET**

**-------------------------------------- TO BE COMPLETED BY RESEARCHER --------------------------------------**

PARTICIPANT INFORMATION

Participant Study ID _______________________________________________

Who is administering the questionnaire? _______________________________________________

Date completing questionnaire   

Language used for administration  English

 Punjabi

 Urdu

 Other: ____________________________

Interpreter used  No

 Yes, family or friend

 Yes, other

Who is present at the interview  Partner

 Family member

 Friend

 Other

 None

MEASUREMENTS

Who is taking the measurements? ____________________________________________

Height   cms  Not able to take

Weight   kgs  Not able to take

Waist   cms  Not able to take

**SECTION A BACKGROUND**

*This first section is about your background.*

A1. What is your date of birth?

  

Day Month Year

A2. What is your relationship with the baby your partner is expecting? I am their;

- Natural father
- Adoptive mother/ father
- Foster mother/ father
- Stepmother/ father
- Other: ____________________________________

A3. What best describes your ethnic group or background?

- White; English/Welsh/Scottish/Northern Irish/British
- White; Irish
- Pakistani
- Indian
- Bangladeshi
- White; Polish
- White; Slovakian
- White; Romanian
- White; Czech
- Other White
- White; Gypsy/Roma or Irish traveller
- Chinese
- African
- Caribbean
- Mixed White and Black Caribbean
- Mixed White and Black African
- Mixed White and Asian
- Any other mixed/multiple ethnic background: ________________________________________
- Any other ethnic background: ________________________________________

A4. What country were you born in?

 England  Northern Ireland  Poland

 Pakistan  Scotland  Czech Republic

 Bangladesh  Wales  Slovakia

 India  Republic of Ireland  Romania

 Hungary

 Other: ________________________

A5. If you were not born in the UK, how old were you when you moved to the UK?

 years old 🞏 Don’t know

A6. What country were your parents born in?

Your mother: Your father:

  England

  Pakistan

  Bangladesh

  India

  Northern Ireland

  Scotland

  Wales

  Republic of Ireland

  Slovakia

  Czech Republic

  Poland

  Romania

  Hungary

  Other: __________________________________________________

  Don’t know

A7. What is your religion?

- None
- Christian (including Church of England, Catholic, Protestant and all Christian denominations)
- Islam
- Sikhism
- Buddhism
- Hinduism
- Judaism
- Any other religion: ________________________________________

A8. How often, if at all, do you pray, or attend services or meetings connected with your religion?

- Every day or more
- Not every day but at least once a week
- Less often than once a week but at least once a month
- Sometimes but less than once a month
- Very rarely or never

**SECTION B HOUSE**

*This next section is about the house you live in at the moment and your previous home.*

B1. If different from the mother of the baby, what is your postcode?  

B2. Do you own or rent your home, or have some other arrangement?

- own with help of mortgage or loan
- own outright
- rent it
- live here rent free (including rent free in relatives’/friends’ property but not squatting)
- living in relatives'/friends' property and paying board
- pay rent and part mortgage (shared ownership)
- squatting
- don’t know

B3. If you are renting your home, who is your landlord?

- Private landlord or letting agency
- Housing association, housing co-operative, charitable trust
- Local authority, local council
- Relative or friend (before you lived here) of a household member
- Employer (individual) of a household member
- Employer (company) of a household member
- Another organisation
- Don’t know

B4. What was the main reason you moved from your last home?

- Needing a bigger/better home or a home in a better area
- Relationship breakdown
- Moving in with partner and/or moving out of parents’ home
- Needed to live closer to my/my partner’s work
- Wanted to live closer to friends/relatives
- Moved to Bradford from abroad
- End of rental agreement last property (contract was up)
- Issues/arguments with previous landlord
- Issues/arguments with neighbours
- Issues/arguments with family/partner/ housemates living in previous home
- Other
- Not applicable; never moved from first home

B5. What is the postcode of your previous home?

 

- Not applicable; moved here from abroad
- Don’t know

B6. How often did you move in the past 5 years?

 times

B7. Are you planning to move house in the next year?

- Yes
- No
- Don’t know

**SECTION C NEIGHBOURHOOD**

*This next section is about the neighbourhood you live in.*

C1. How satisfied or dissatisfied are you with the area you live in?
*By your area, I mean within about a mile or 20 minute walk of your home.*

- Very satisfied
- Fairly satisfied
- Neither satisfied nor dissatisfied
- Fairly dissatisfied
- Very dissatisfied

C2. How satisfied or dissatisfied are you with the parks and green spaces in your local area?

- Very satisfied
- Fairly satisfied
- Neither satisfied nor dissatisfied
- Fairly dissatisfied
- Very dissatisfied

C3. How often do you visit Bradford’s parks and green spaces?

During the winter months (September – March) During spring and summer (April - August)

- 5 times a week or more  5 times a week or more
- 2 to 4 times a week  2 to 4 times a week
- once a week  once a week
- 1 to 3 times a month  1 to 3 times a month
- less than once a month  less than once a month

C4. Thinking about your neighbourhood, to what extent do you agree with this statement:

*Other people think this is a good area.*

- Strongly agree
- Agree
- Neither agree nor disagree
- Disagree
- Strongly disagree

C5. Do children in your area have an outdoor space or facilities nearby where they can play safely?

- Yes
- No
- Don’t know

**SECTION D LANGUAGE**

*This next section is about languages you speak, languages in your households and books in your home.*

D1. What is your first language?

*Please tick two boxes if you are bilingual (you speak two languages fluently and were taught these languages from a young age)*

- English
- Punjabi
- Urdu (including Hindi)
- Polish
- Slovakian
- Hungarian
- Romanian
- Russian
- Gujarati
- Spanish
- Pashto
- Bengali (including Sylheti, Chatgaya/Chittagonian)
- Arabic
- Other: ______________________

*If there are no children living in your home please skip the next question and go to D3.*

D2. If there are children living in your home already, which languages do they speak?

- English
- Punjabi
- Urdu (including Hindi)
- Polish
- Slovakian
- Hungarian
- Romanian
- Russian
- Gujarati
- Spanish
- Pashto
- Bengali (including Sylheti, Chatgaya/Chittagonian)
- Arabic
- Other: ______________________

*If English is your first language please skip the next question and go to D4.*

D3. If English is not your first language, how well can you do the following things **in English**:

**Not at all A little bit Some Quite well Very well**

Listening     

Reading     

Writing     

Speaking     

D4. How well can you do the following things **in your first language**:

*If you are bilingual choose the language you are most comfortable with.*

**Not at all A little bit Some Quite well Very well**

Listening     

Reading     

Writing     

Speaking     

D5. With which statement do you agree the most? Please tick only one box.

- It is important to me that my child does learn English before starting school
- It does not matter to me
- It is important to me that my child **does not** learn English before starting school

D6. How often do you read books for pleasure?

- (Almost) never
- Once or twice a month
- About once a week
- Several times a week
- Daily

D7. If you have children with your current partner who are of preschool age or in primary school, how many days in a typical week do you read with them?

- 0
- 1
- 2
- 3
- 4
- 5
- 6
- 7

**SECTION E EDUCATION**

*This next section is about your education. Please skip the questions that don’t apply to you.*

E1. Where did you obtain your highest educational qualification?

- England
- Pakistan
- India
- Bangladesh
- Poland
- Slovakia
- Hungary
- Elsewhere

E2. If your highest educational qualification was obtained in **England**, what is the highest level you completed? *Please tick only one box.*

- Any GCSE, SCEs (less than 5 GCSEs grades A-C)
- 5 or more GCSEs (grades A-C)
- GNVQ foundation level
- NVQ1
- GNVQ intermediate
- NVQ 2
- (Young) apprenticeship
- NVQ 3
- Advanced apprenticeship
- GNVQ Advanced
- AS or A level, International Baccalaureate or BTEC
- NVQ 4/5
- Higher National Certificate/ Higher National Diploma, Higher Education Diploma
- Foundation Degree
- Bachelor’s degree 3-5 years
- Master’s degree (taught/research) or Postgraduate qualification, Doctorate/ PhD
- Other: _______________________________
- None of these qualifications
- Don’t know

E3. If your highest educational qualification was obtained in **Pakistan**, what is the highest level you completed? *Please tick only one box.*

- Secondary School Certificate/ Matriculation
- Higher Secondary or Intermediate Certificate
- Technical Education Certificate/ Diploma
- Registered Nurse/ General Nursing Diploma
- Primary Teaching Certificate
- Certificate in Training, Paramedic Secondary School Certificate/ Matriculation
- Bachelor Degree Arts/Sciences, Education, Engineering, Medicine/Surgery
- Master Degree/ Master of Education/ Master of Philosophy (MPhil), Doctorate
- Other: ________________________________
- None of these qualifications
- Don’t know

E4. If your highest educational qualification was obtained in **India**, what is the highest level you completed?

*Please tick only one box.*

- Senior Secondary School Leaving Certificate
- Matriculation Certificate, Senior School Certificate, ITI Certificate
- Diploma in Technical Education, Junior basic teacher’s training, Nursing Diploma/Certificate
- Bachelor’s Degree, Bachelor of Law/ Education
- Master’s Degree, Master of Philosophy (Mphil), Doctor of Philosophy/ Letters
- Other: ________________________________
- None of these qualifications
- Don’t know

E5. If your highest educational qualification was obtained in **Bangladesh**, what is the highest level you completed? *Please tick only one box.*

- Secondary School/ Higher Secondary Certificate
- Dakhil/ Alim Certificate
- SSC Vocational/ Trade Certificate
- HSC Business Management
- HSC Vocational
- Diploma in Commerce Certificate
- Diploma in Engineering
- Certificate in Education (CinEd)
- Diploma in Agriculture/Ayurvedic & Unanai Medicine/ Textile/ Health Technology/ Survey/ Ceramics/ Nursing/ Graphic Arts
- Bachelor Degree Pass, Bachelor Degree
- Master’s Degree, PGD and PhD
- Fazil/ Kamil Degree
- Other: ________________________________
- None of these qualifications
- Don’t know

E6. If your highest educational qualification was obtained in **Poland**, what is the highest level you completed? *Please tick only one box.*

- General Secondary School Leaving Certificate/ Diploma
- Technikum, Liceum Mature Certificate/Diploma
- Technical Secondary School, Specialised Secondary School
- General/ Supplementary Secondary School
- Ballet School Diploma (technician level), Second level music school Diploma, Circus School Diploma (technician level)
- Basic vocational School
- Post-secondary School Certificate/ Diploma
- Teacher Training, Diploma Social Work College
- 1st Degree Studies
- University Studies, Magister/Lekarz, Postgraduate Certificate
- Master’s Degree, Doctoral Study/PhD
- Other: ________________________________
- None of these qualifications
- Don’t know

E7. If your highest educational qualification was obtained in **Slovakia**, what is the highest level you completed? *Please tick only one box.*

- Study of selected subjects (Štúdium jednotlivých predmetov)
- Vocational School (Odborné učilište)
- Secondary Specialised School without maturita (Vysvedčenie o záverečnej skúške, Výučný list)
- Retraining Courses Certificate (Osvedčenie)
- Secondary Specialised School with maturita (Vysvedčenie o maturitnej skúške, Vysvedčenie o maturitnej skúške, Výučný list)
- Gymnasium (gymnázium)
- Follow-up courses (Nadstavbové štúdium)
- Post-secondary Qualification Study (Pomaturitné kvalifikačné štúdium)
- Supplementary Pedagogical Study (Doplňujúce pedagogické štúdium)
- Post-secondary Specialised Study (Pomaturitné špecializačné štúdium)
- Conservatoire (Konzervatórium)
- Higher Professional Studies/ Graduate Diploma (Absolventský diplom), Bachelor
- Master, Magister, Doctoral, Engineer, PhD, Extensive study for teaching diploma
- Other: ________________________________
- None of these qualifications
- Don’t know

E8. If your highest educational qualification was obtained in **Hungary**, what is the highest level you completed? *Please tick only one box.*

- Certification of the Maturity Examination (Párhuzamos oktatás szakközépiskoláb)
- Upper Secondary General School
  ( Gimnázium)
- Upper Secondary Vocational School (Szakközépiskola)
- Vocational certificate based on NVQL examination (Párhuzamos oktatás szakiskolában, Szakiskola, előkészítő szakiskola, Alapfokú iskolai végzettségre épülő szakképzés, évfolyamra épülő szakképzés)
- Vocational certificate based on NVQL examination (Szakképző évfolyamok középiskola utolsó évfolyamára vagy érettségire épülő nem felsőfokú OKJ szakmákban)

- Tertiary Vocational Program (Felsőfokú szakképzés)
- Bachelor’s Degree (Alapképzés), Master’s Degree (Osztatlan képzés/ Master), University Diploma (Egyetemi szintű alapképzés), College Diploma (Főiskolai szintű alapképzések), Certificate in Specialisation (Szakirányú továbbképzés), Doctorate/PhD
- Other: _____________________________
- None of these qualifications
- Don’t know

E9. If your highest educational qualification was obtained elsewhere, what is the highest level you completed?

Country: _______________________________________________________________

Type of school/institution: _______________________________________________________________

Educational programme: _______________________________________________________________

Degree/qualification: _______________________________________________________________

**SECTION F SOCIOECONOMIC CIRCUMSTANCES**

*This section is about your socioeconomic circumstances, such as your employment and financial situation.*

F1. Are you currently a full-time student?

- Yes  No

F2. Are you currently employed?

- Yes  No

F3. If you are not currently employed, have you ever worked before?

- Yes  No; *please go to question F12.*

F4. If you have worked before but you are not working right now, how long ago have you stopped working?

 years and  months ago. Or if less than a month ago:  weeks ago.

F5. Are you currently on sick leave?

- Yes  No

*Please answer the following questions about your current job, or about your previous job if you are not working at the moment.*

F6. Did/do you work as an employee or are/were you self-employed?

- Employee
- Self-employed with employees (go to question F8)
- Self-employed/freelance without employees (go to question F9)
- Student in Training (apprentice)

F7. How many people work/worked for your employer at the place where you work/worked?

- Work alone
- 1 - 5 people
- 6 - 24 people
- 25 – 49
- 50-499
- 500 or more

F8. If you are self-employed, how many people do (did) you employ?

- 1-24  25 or more

F9. Do (did) you supervise any other employees? (A supervisor or foreman is responsible for overseeing the work of other employees on a day-to-day basis)

- Yes  No

F10. How many hours did or do you work in a typical week?

  hours a week

F11. Which of these best describes the sort of work you do/did?

- Modern professional occupations such as: teacher – nurse - physiotherapist – social worker – welfare officer – artist– musician – police officer (sergeant or above) – software designer
- Clerical and intermediate occupations such as: secretary – personal assistant – clerical worker – office clerk – call centre - agent – nursing auxiliary – nursery nurse
- Senior managers or administrators (usually responsible for planning, organising and co-ordinating work, and for finance) such as: finance manager – chief executive
- Technical and craft occupations such as: motor mechanic - fitter – inspector – plumber – printer – tool maker – electrician – gardener – train driver
- Semi-routine manual and service occupations such as: postal worker – machine operative – security guard – caretaker - farm worker – catering assistant – receptionist – sales - assistant
- Routine manual and service occupations such as: HGV driver – van driver – cleaner – porter – packer – sewing machinist – messenger – labourer – waiter/waitress – bar staff
- Middle or junior managers such as: office manager – retail manager – bank manager – restaurant manager – warehouse manager – publican
- Traditional professional occupations such as: accountant - solicitor – medical practitioner – scientist – civil/mechanical engineer
- Other: ________________________________________________________________

F12. Do you (and your partner) receive any of these benefits/ credits/ allowances?

- Child Benefit
- Child Tax Credit
- Childcare Grant
- Healthy Start
- Maternity Allowance
- Working Tax Credit
- Income Support
- Disability Living Allowance
- Income-based Jobseekers’ allowance
- Income-related Employment and Support allowance
- Housing benefit/ rent rebate/ council tax benefit
- Local Housing Allowance
- Incapacity Benefit
- Pension Credit
- Carer’s Credit
- Universal Credit
- Other: ____________________________________________
- None
- Don’t know
- Does not wish to answer

F13. This table shows income in weekly, monthly and annual amounts. Which of the amounts on this list represents the **total income of your household**; income from all jobs, (full and part time), all tax credits, all benefits and all other sources and earnings after tax when all income is added together.
*Please tick only one box.*


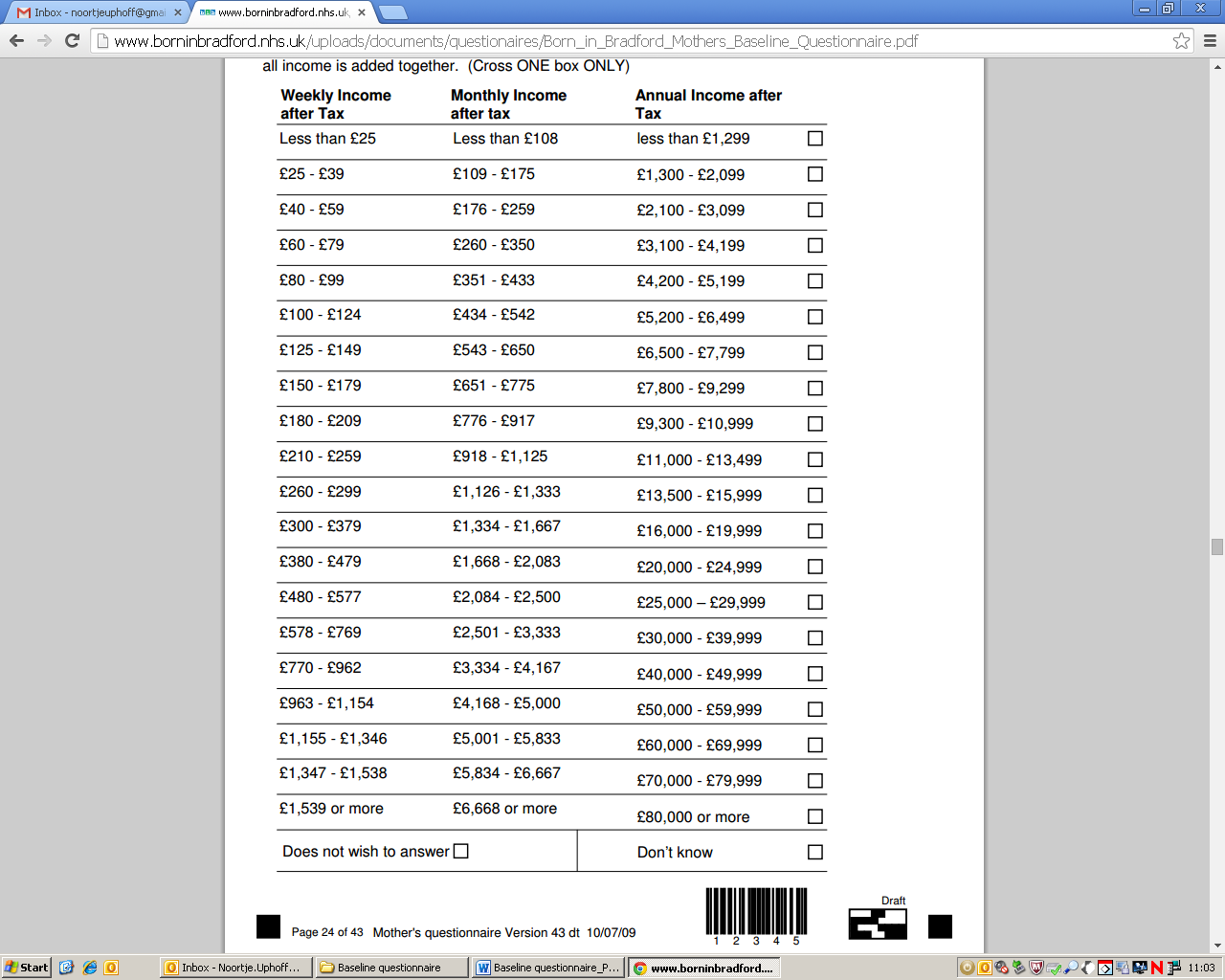


F 14. Who manages the finances in the household? Tick the box for everyone who manages the household budget, makes decisions about large purchases, pays bills, applies for benefits/tax credit/loans and so on.

- Me
- My partner
- My mother
- My father
- Mother in law
- Father in law
- Someone else: ___________________________
- Don’t know
- Does not wish to answer

F 15. This question asks about financial resources of household. Do you (and your partner) have:

|  | **Yes** | **No, I would like to but cannot afford this at the moment** | **No, I do not want or need this at the moment** | **No, other reason** | **Do not wish to answer** |
| --- | --- | --- | --- | --- | --- |
| Drinks or a meal with friends or family who don’t live with you at least once a month |  |  |  |  |  |
| Money to make regular savings of £20 a month or more for rainy days or retirement |  |  |  |  |  |
| Money to replace any worn out furniture |  |  |  |  |  |
| Money to replace or repair major electrical goods |  |  |  |  |  |
| A small amount of money to spend on yourself each week |  |  |  |  |  |
| In winter are you able to keep your home warm enough |  |  |  |  |  |
| Fresh fruit and vegetables on a daily basis |  |  |  |  |  |

F 16. How well would you say you (and your partner) are managing financially these days?
Would you say you are:

- Living comfortably
- Doing alright
- Just about getting by
- Finding it quite difficult
- Finding it very difficult

- Don’t know
- Does not wish to answer

F 17. Compared to a year ago, how would you say you (and your partner) are doing financially now?

- Better off
- About the same
- Worse off
- Don’t know
- Does not wish to answer

F 18. In the past year have you or you and your partner taken a loan or credit with a short term loan company (e.g. payday loan, BrightHouse, Wonga) to pay for items such as clothing, furniture, phone?

- No
- Yes – 1 time
- Yes – 2 or more times
- Don’t know
- Does not wish to answer

F 19. In the past year have family, friends or relatives lent you or you and your partner money
to pay for items such as clothing, furniture, phone?

- No
- Yes – 1 time
- Yes – 2 or more times
- Don’t know
- Does not wish to answer

F 20. Sometimes people are not able to pay every bill when it is due. Are you up to date with all bills?

- Yes
- No
- Don’t know
- Does not wish to answer

F 21. If you are not up to date with some bills, which ones are you behind with?

- Electricity, Gas or other household
  fuel bills
- Council tax
- Insurance Policies
- Mobile or home telephone, internet or television
- Water rates
- Credit cards (bank or store credit cards)
- Short term loans/credit (e.g. payday loan, brighthouse)
- Other bills

_____________________________

- Don’t know
- Does not wish to answer

*Think of this ladder as showing where people stand* ***in your neighbourhood****. By your neighbourhood, I mean within about a mile or 20 minute walk of your home.*

*At the top of the ladder are the people who are the best off – those who have the most money, the best education, and the most respected jobs. At the bottom are the people who are the worst off – who have the least money, least education, and the least respected job or no job.*

*The higher up you are on this ladder, the closer you are to the people at the top; the lower you are, the closer you are to the people at the bottom.*

F 22. Where would you place yourself on this ladder? Please tick the box next to the rung where you think you stand at this time of your life relative to other people **in your neighbourhood**.


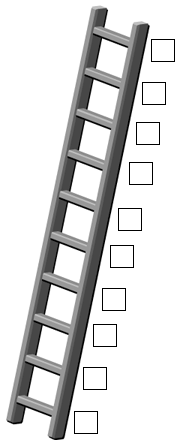


 Does not wish to answer

You have just ranked yourself on the social ladder compared to other people in your neighbourhood. Could you now do the same, but with the ladder representing all people **in England**?

The higher up you are on this ladder, the closer you are to the people at the top; the lower you are, the closer you are to the people at the bottom.

F 23. Where would you place yourself on this ladder? Please tick the box next to the rung where you think you stand at this time of your life relative to other people **in England**.


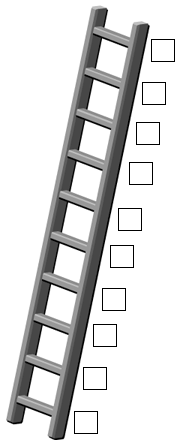


 Does not wish to answer

**SECTION G SOCIAL CIRCUMSTANCES**

*This section is about your social circumstances and activities you take part in in the community.*

G1. How often do you feel that you, personally, have been discriminated against
because of your ethnicity, race, or religion?

- Never  Does not wish to answer
- Rarely  Don’t know
- Sometimes
- Often

G2. Generally speaking, would you say that most people can be trusted or you can’t be too careful in dealing with people?

- Can be trusted  Don’t know
- 2  Do not wish to answer
- 3
- 4
- Can’t be too careful

G3. Do you regularly join in the activities of any organisations or clubs?

- Yes
- No

G4. If yes, do you participate in any of these organisations?

- Voluntary community work (such as food bank)
- Environmental group
- Political party
- Trade union
- Parent-teacher association
- Tenants group
- Attending a religious group, church or mosque
- Youth group
- Social club
- Sports club
- Other: _________________________________

**SECTION H HEALTH AND WELLBEING**

*This is the last section of the questionnaire. It is about your health and wellbeing, and includes topics such as health behaviour, mental health and your feelings about the baby.*

H1. How would you describe your health generally?

- Excellent
- Very good
- Good
- Fair
- Poor
- Don’t know
- Does not wish to answer

H2. How would you describe the health of your teeth and mouth?

- Excellent
- Very good
- Good
- Fair
- Poor
- Don’t know
- Does not wish to answ

H3. Do you regularly smoke cigarettes at the moment (not e-cigarettes)?

- Yes
- No
- Does not wish to answer

H4. If yes, about how many cigarettes do you smoke a day on average?

- Less than 1 a day (only smoke occasionally)
- 1-5 cigarettes
- 6-10 cigarettes
- 11-20 cigarettes
- Over 20 cigarettes

H5. Do you drink alcohol, for example beer, wine or spirits?

- Yes
- No
- Does not wish to answer

H6. If yes, what is the weekly average number of units you drink?

Please add up all the units of any sort of alcohol you drink in a typical week and enter in the box(es) provided.

 units of alcohol a week

*Now we would like to ask you a few questions about your mental health.*

H7. Over the last 2 weeks, on how many days have you been bothered by any of the following problems?

|  | **Not at all** | **Several days** | **More than half the days** | **Nearly every day** | **Do not wish to answer** |
| --- | --- | --- | --- | --- | --- |
| 1. Little interest or pleasure in doing things |  |  |  |  |  |
| 2. Feeling down, depressed, or hopeless |  |  |  |  |  |
| 3. Trouble falling or staying asleep, or sleeping too much |  |  |  |  |  |
| 4. Feeling tired or having little energy |  |  |  |  |  |
| 5. Poor appetite or overeating |  |  |  |  |  |
| 6. Feeling bad about yourself — or that you are a failure or have let yourself or your family down |  |  |  |  |  |
| 7. Trouble concentrating on things, such as reading the  newspaper or watching television |  |  |  |  |  |
| 8. Moving or speaking so slowly that other people could have noticed? Or the opposite — being so fidgety or restless that you have been moving around a lot more than usual? |  |  |  |  |  |
| 9. Thoughts that you would be better off dead or of hurting yourself in some way |  |  |  |  |  |

 Do not wish to answer these questions

H8. If you checked off any problems, how difficult have these problems made it for you to do your work, take care of things at home, or get along with other people?

- Not difficult at all
- Somewhat difficult
- Very difficult
- Extremely difficult

H9. What kind of milk do you usually use for drinks in tea or coffee and on cereals etc?

- whole milk
- semi-skimmed
- skimmed
- other kind (please specify)
- no usual type
- don’t know
- do not drink milk

H10. Do you usually take sugar in:

Yes No

Tea  

Coffee  

- do not drink tea/coffee

H11. Which type of breakfast cereal do you normally eat?

- high fibre (eg All Bran, Branflakes, Shredded Wheat, Muesli, Porridge, Weetabix
- other (eg Cornflakes, Rice Krispies, Special K, Sugar Puffs, Honey Snacks
- no usual type
- do not eat breakfast cereal


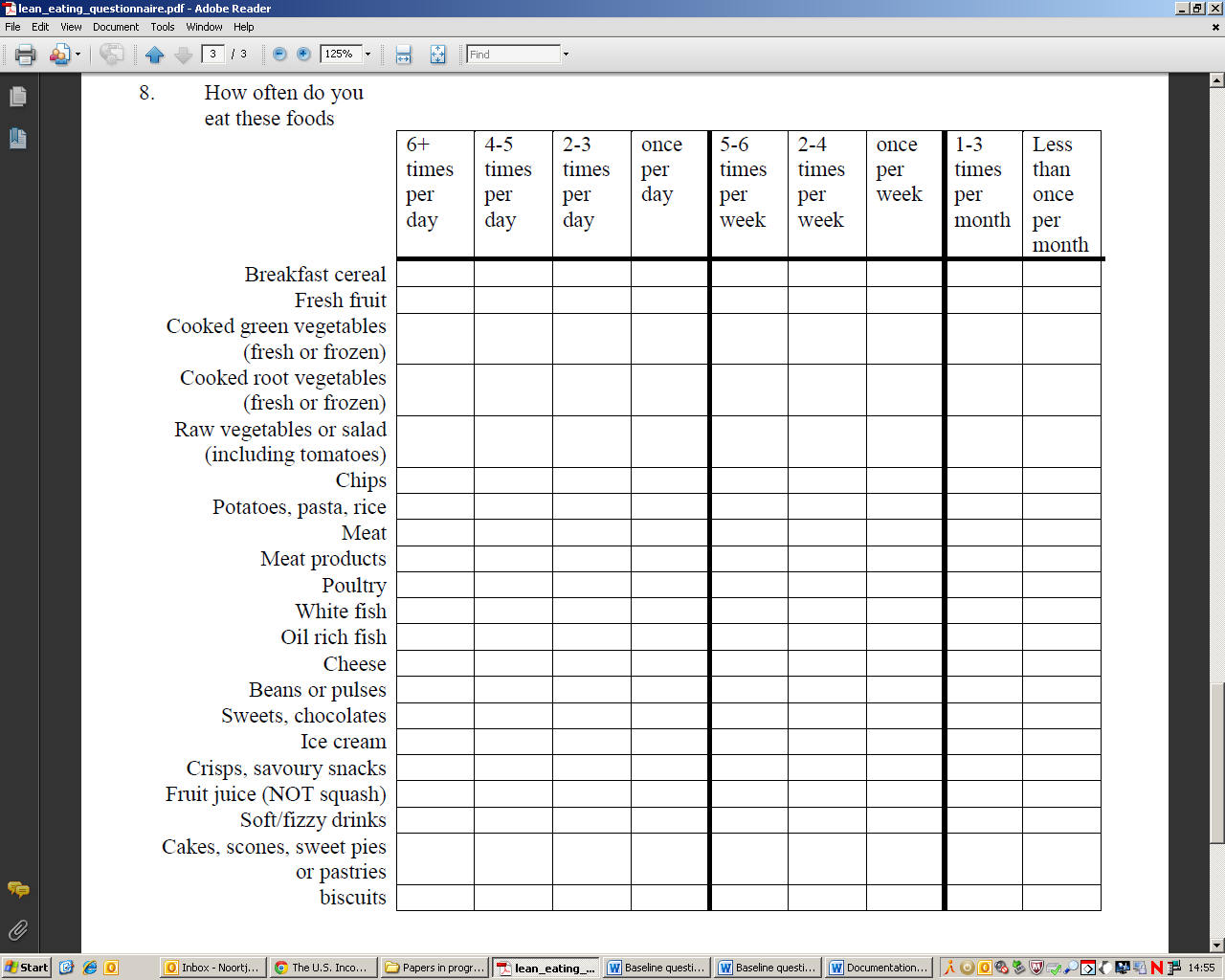


H12. How often do you eat these foods:

H13. Over the last 2 weeks, on how many days have you been bothered by any of the following problems?

|  | **Not at all** | **Several days** | **More than half the days** | **Nearly every day** | **Do not wish to answer** |
| --- | --- | --- | --- | --- | --- |
| 1. Feeling nervous, anxious or on an edge? |  |  |  |  |  |
| 2. Not being able to stop or control worrying? |  |  |  |  |  |
| 3. Worrying too much about different things? |  |  |  |  |  |
| 4. Trouble relaxing? |  |  |  |  |  |
| 5. Being so restless that it is hard to sit still? |  |  |  |  |  |
| 6. Becoming easily annoyed or irritable? |  |  |  |  |  |
| 7. Feeling afraid as if something awful might happen? |  |  |  |  |  |

**SECTION I BABY AND PREGNANCY**

*Below are some questions that ask about your circumstances and feelings around the time your partner became pregnant with this baby. By ‘your partner’ we mean the mother of the baby.*

I1. In terms of becoming a father (first time or again), I feel that this pregnancy happened at the…
*(please tick the statement which most applies to you)*

- right time
- ok, but not quite right time
- wrong time

I2. Just before my partner became pregnant…
*(please tick the statement which most applies to you)*

- I intended for her to get pregnant
- my intentions kept changing
- I did not intend for her to get pregnant

I3. Before my partner became pregnant…
*(please tick the statement which most applies to you)*

- my partner and I had agreed that we would like her to be pregnant
- my partner and I had discussed having children together, but hadn’t agreed for her to get pregnant
- we never discussed having children together

*These questions are about your thoughts and feelings about the developing baby.*

*Please tick one box only in answer to each question.*

I4. Over the past two weeks I have thought about,
or been preoccupied with the developing baby:

- Almost all the time
- Very frequently
- Frequently
- Occasionally
- Not at all

I5. Over the past two weeks when I have spoken about, or thought about the developing baby I got emotional feelings which were:

- Very weak or non-existent
- Fairly weak
- In between strong and weak
- Fairly strong
- Very strong

I6. Over the past two weeks my feelings about
the developing baby have been:

- Very positive
- Mainly positive
- Mixed positive and negative
- Mainly negative
- Very negative

I7. Over the past two weeks I have had the desire to read about or get information about the developing baby. This desire is:

- Very weak or non-existent
- Fairly weak
- Neither strong nor weak
- Moderately strong
- Very strong

I8. Over the past two weeks I have been trying
to picture in my mind what the developing baby actually looks like in my partner’s womb:

- Almost all the time
- Very frequently
- Frequently
- Occasionally
- Not at all

I9. Over the past two weeks I think of the developing baby mostly as:

- A real little person with special characteristics
- A baby like any other baby
- A human being
- A living thing
- A thing not yet really alive

I10. Over the past two weeks when I think about
the developing baby, my thoughts:

- Are always tender and loving
- Are mostly tender and loving
- Are a mixture of both tenderness and irritation
- Contain a fair bit of irritation
- Contain a lot of irritation

I11. Over the past two weeks my ideas about possible names for the baby have been:

- Very clear
- Fairly clear
- Fairly vague
- Very vague
- I have no idea at all

I12. Over the past two weeks when I think about
the developing baby I get feelings which are:

- Very sad
- Moderately sad
- A mixture of happiness and sadness
- Moderately happy
- Very happy

I13. Over the past two weeks I have been thinking about what kind of child the baby will grow into:

- Not at all
- Occasionally
- Frequently
- Very frequently
- Almost all the time

I14. Over the past two weeks I have felt:

- Very emotionally distant from the baby
- Moderately emotionally distant from the baby
- Not particularly emotionally close to the baby
- Moderately close emotionally to the baby
- Very close emotionally to the baby

I15. When I first see the baby after the birth I expect I will feel:

- intense affection
- mostly affection
- affection, but I expect there may be a few aspects of the baby I will dislike
- I expect there may be quite a few aspects of the baby I will dislike
- I expect I might feel mostly dislike

I16. When the baby is born I would like to hold
the baby:

- Immediately
- After it has been wrapped in a blanket
- After it has been washed
- After a few hours for things to settle down
- The next day

I17. Over the past two weeks I have had dreams
about the pregnancy or baby:

- Not at all
- Occasionally
- Frequently
- Very frequently
- Almost every night

I18. Over the past two weeks I have found myself feeling, or rubbing with my hand, the outside of
my partner's stomach where the baby is:

- A lot of times each day
- At least once per day
- Occasionally
- Once only
- Not at all

 Do not wish to answer these questions

This is the end of the questionnaire.

If you have any questions about our study, please ask the researcher.

Thank you very much for your help!

**Appendix 5: Details of the collection of Biological Samples**

**5.1 Additional samples for the BiBBS cohort Biological Bank**

Additional biological samples will be collected at recruitment and shortly after birth, for immediate analysis and for storage within the biobank. The biobank will be stored in the BiB biobank, which has been set up and maintained at BIHR.

*For the women:* Women who are recruited at their GTT will have fasting blood taken (to measure insulin, glucose, and lipid profile) along with 19 mls of blood and 21 mls urine, for storage at -80^0^C in the biological bank (see below). Women who are recruited at another time will not have the fasting sample taken.

All blood samples for storage will be collected into standard 4–9 mL tubes. Four are collected prior to the start of the GTT test to obtain fasting blood; these are: 2 x 4 mL gel tubes, 1 x 9 mL EDTA tube and 1 x 4 mL sodium fluoride oxalate. At the end of the GTT test, 2 hours post administration of the polycal drink, a blood sample is taken into one 4 mL sodium fluoride oxalate tube.

Tubes are drawn at the GTT in the order shown in Table 3.3.

**Table 5.1: Blood samples at the GTT for routine clinical use and to store in the biobank**

| **Tubes** | **To include in BiBBS cohort** |
| --- | --- |
|  | **biobank?** |
| Tube 1 (gel tube) providing serum (2 aliquots) | Yes |
| Tube 2 (gel tube) providing serum for baseline analysis of fasting lipids (cholesterol, | Yes |
| high density lipoproteins (HDL), low density lipoprotein (LDL), triglyceride) and |  |
| derivation of HOMA, an indicator of insulin resistance and beta-cell function |  |
| Tube 3 (EDTA tube) providing whole blood (one aliquot as a source of genomic | Yes |
| DNA), plasma (three aliquots), buffy coat (one aliquot) and red blood cells (one |  |
| aliquot) |  |
| Tube 4 (sodium fluoride oxalate) used to derive fasting glucose levels | No – routine sample |
|  |  |
| Tube 5 (sodium fluoride oxalate) used to measure glucose response 2 hours after | No – routine sample |
| administration of a polycal (sugary) drink |  |

Tubes 1 and 3 are sent to the Clinical Biochemistry Laboratory together for processing as a set of BiBBS cohort samples; these blood samples, as well as the urine sample, are collected directly from the antenatal clinic by the a member of the research team.

Tubes 2, 4 and 5 are processed in the Clinical Biochemistry Laboratories according to standard protocol; these samples are handled by the standard portering system.

At the GTT we will collect fasting blood. This allows the greatest range of tests to be conducted, including lipids. If women do not attend the GTT, we will be unlikely to be able to collect fasting blood. We will still ask to collect a (non-fasted) blood sample for the biobank – this will prohibit some of the tests, but many can still be conducted.

Urine

A single void urine sample (collected at baseline) of ~ 50 mL is collected into sterile specimen containers and three aliquots of 7ml stored at -80^0^C in the biobank.

Hair

A small strand of hair will be cut from the back of the mother‟s head shortly after birth. This will be conducted by midwives/trained researchers, according to a written protocol (developed and implemented for the HELIX study). The end of the hair that was closest to the scalp will be marked. We will provide training to midwives on the process.

*For the babies*: Umbilical cord blood will be collected for the biobank at birth using a large syringe and a 20 gauge needle. One 4 mL gel tube (Tube 1) and one 9ml EDTA tube (Tube 2) are collected. Tube 1 will provide serum (two aliquots) for biobanking; tube 2 will provide whole blood (one aliquot as a source of genomic DNA), plasma (three aliquots), buffy coat (one aliquot) and red blood cells (one aliquot).

*For the partners:* Partners will be asked to provide a single saliva sample at a convenient time (such as at the maternal GTT assay, or at birth of the baby), for storage at room temperature.

**5.2 Biological samples: storage and tracking**

A number of principles will be established with respect to the biological samples described above. First, the samples should be processed in such a way as to enable the widest possible range of analytical tests to be conducted. This is in recognition of the fact that it is not possible to predict from the outset of a study the scope of specific analyses, as technologies and tests available change. Second, the protocol seeks a balance between the ideal intensity of sampling for scientific purposes and the practical and logistic setting in which the work is conducted. Third, samples are to be processed in a timely fashion and there should be an avoidance of freeze-thaw cycles. Fourth, cataloguing and storage of samples will be subject to a secure and robust inventory system. Fifth, best practice will be followed to maintain the highest standard in clinical and research governance, including fulfilling our obligations and responsibilities laid down in the ethical and human tissue authority legislation.

Once the samples have been collected, according to the procedures detailed in section 3.7 above, the Biobank has a system for recording the exact location of each sample and its storage conditions. All equipment failures or freeze/thaw episodes will be recorded. Review of all storage equipment will occur annually. All samples will be collected and processed following Standard Operating Procedures. For each specimen the time from collection to storage is recorded on a laboratory information management system (LIMS). All aliquots and samples will have a unique code to allow sample tracking. Consent for samples to be stored, and indeed where consent has been revoked, will be tracked. This will be done through direct linkage between the project management IT system and the LIMS. A procedure is in place for disposing of samples when sample consent is removed, or has not been collected in a suitable time-frame.

The LIMS permits the following: samples to be logged on arrival to the laboratory; samples to be linked with the cohort main database; derivatives from the sample to be recorded and stored; each piece of material (e.g. a tube of DNA or a paper of dried blood) to be tracked with a unique identifier; consent to be monitored in real-time to maintain compliance with the HTA guidelines.

The biobank has clear guidelines for access to and use of samples, and the Born in Bradford Executive group will act as custodians (see section 8.1.3). This group is responsible for oversight of clinical and laboratory quality, for safety, and for assessing requests for sample access.

**Appendix 6 Data capture**

There are four types of data that will be captured from various sources as set out in Table 4.1, below.

**Table 6.1: Cohort data capture: type, source, and flow.**

|  | **Data Type** |  | **Data** |  | **Identity** |  | **Capture** |  | **Data Flow** |  |  |  |
| --- | --- | --- | --- | --- | --- | --- | --- | --- | --- | --- | --- | --- |
|  |  |  |  |  |  |  |  |  |  |  |  |  |
|  |  |  | **Source** |  |  |  |  |  |  |  |  |  |
| **Primary** | |  | Demographics |  | NHS no. |  | Registration form |  | Initial validation of registration form data via | |  |  |
|  |  |  |  |  |  |  | *AND* PDS^a^ |  | PDS. Subsequent 1-monthly PDS data | |  |  |
|  |  |  |  |  |  |  |  |  | request to track participants. | |  |  |
|  |  |  | Baseline |  | BSB Person |  | Tablet-based |  | Automated upload to cohort RDBMS^f^. Early | |  |  |
|  |  |  | questionnaire |  | ID *AND* BSB |  | electronic |  | paper-based versions may be used. Upon | |  |  |
|  |  |  |  |  | Pregnancy |  | questionnaire |  | deployment of the electronic version, these | |  |  |
|  |  |  |  |  |  |  |  |  | will be backfilled by double entry. | |  |  |
|  |  |  |  |  | b |  |  |  |  |  |  |  |
|  |  |  |  |  | no. |  |  |  |  |  |  |  |
|  |  |  | Clinic |  | BSB Person |  | Tablet-based |  | Automated upload to cohort RDBMS. Early | |  |  |
|  |  |  |  |  | ID *AND* BSB |  | electronic |  | paper-based versions may be used. Upon | |  |  |
|  |  |  |  |  | Pregnancy no. |  | questionnaire |  | deployment of the electronic version, these | |  |  |
|  |  |  |  |  |  |  |  |  | will be backfilled by double entry. | |  |  |
|  |  |  |  |  |  |  |  |  |  |  |  |  |
| **Data** | |  | Maternity |  | NHS no. |  | BTHFT^c^ routine |  | Obtained via 6-monthly BTHFT data request | |  |  |
| **Linkage** | |  | hospital |  |  |  | data |  | then uploaded to cohort RDBMS | |  |  |
|  |  |  |  |  |  |  |  |  |  |  |  |  |
|  |  |  | Primary care |  | NHS no. |  | SystmOne (TPP) |  | Obtained via 6-monthly TPP data request | |  |  |
|  |  |  |  |  |  |  | routine GP data |  | then uploaded to cohort RDBMS | |  |  |
|  |  |  | Pathology |  | Hospital no. |  | BTHFT routine |  | Obtained via 12-monthly BTHFT data | |  |  |
|  |  |  |  |  | UPN^d^ |  | data |  | request then uploaded to cohort RDBMS | |  |  |
|  |  |  | Education |  |  |  | Local Authority |  | Obtained via 4-monthly Local Authority data | |  |  |
|  |  |  | HES^e^ |  |  |  | routine data |  | request then uploaded to cohort RDBMS | |  |  |
|  |  |  |  |  | NHS no. |  | HSCIC routine |  | Obtained via biennial HSCIC data request | |  |  |
|  |  |  |  |  |  |  | data |  | then uploaded to cohort RDBMS | |  |  |
| **Project** | |  | Contacts and |  | NHS no. |  | SystmOne |  | Obtained by running quarterly reports in | |  |  |
|  |  |  | outcomes |  |  |  |  |  | SystmOne then uploaded to cohort RDBMS | |  |  |
|  |  |  |  |  | NHS no. *OR* |  | MS Access Forms |  | Obtained via quarterly Project centre data | |  |  |
|  |  |  |  |  | BSB Person |  |  |  | request then uploaded to cohort RDBMS | |  |  |
|  |  |  |  |  | ID |  |  |  |  |  |  |  |
| **Biosample** | |  | Tracking |  | Aliquot |  | Web and tablet |  | Automated upload to cohort RDBMS. | |  |  |
|  |  |  |  |  | barcode |  | based electronic |  | Barcodes are assigned to aliquots at initial | |  |  |
|  |  |  |  |  |  |  | sample |  | lab processing and thereafter barcode is the | |  |  |
|  |  |  |  |  |  |  |  |  | primary identity, linked to BSB Person ID and | |  |  |
|  |  |  |  |  |  |  | processing |  |  |  |  |  |
|  |  |  |  |  |  |  |  |  | BSB Pregnancy no. in the database | |  |  |
|  |  |  |  |  |  |  | utilities |  |  |  |  |  |
|  |  |  |  |  |  |  |  |  |  |  |  |  |
|  |  |  | Assays |  | Aliquot |  | Procedure |  | Data derived from biosamples in lab assays | |  |  |
|  |  |  |  |  | barcode |  | depends on type |  | will usually be transferred back to the BSB | |  |  |
|  |  |  |  |  |  |  |  |  | team as spreadsheets or csv files via secure | |  |  |
|  |  |  |  |  |  |  |  |  | | | |  |

email then uploaded to cohort RDBMS

of laboratory assay

Explanation of headings: Identify is the unique reference number that will be used to link to the cohort database; Capture is the way that data will be recorded/entered into a system; Data flow is the way that data will be migrated to the cohort database.

^a^PDS = Patient Demographic Service. ^b^For mothers‟ recruitment data, data capture and storage processes will need to index the person *and* the pregnancy. ^c^BTHFT = Bradford Teaching Hospitals NHS Foundation Trust. ^d^UPN = Unique Pupil Number. ^e^HES = Hospital Episode Statistics. ^f^RDBMS = Relational Database Management System.

- - 1. **Data quality and standards**

Primary data captured by electronic forms will be synchronised with the cohort Relational Database Management System (RDBMS) to verify identifiers and validate newly captured data against existing data. Automatic interactive validation will be built into individual data item inputs in electronic forms and questionnaires, such as logical traps, format validations, range limiters, automated questionnaire flow with mandatory values. Backfilled paper forms will be double entered with conflicts assessed by trained operators. Electronic data capture clients for primary data and biosample tracking data will synchronise with the cohort RDBMS, from which unattended transform and report functions will provide regular datasets for researchers to analyse to ensure data quality standards are being met.

Record matches for routine data linkage will be validated on the basis of unique identifiers (e.g. NHS number) plus multiple non-unique identifiers (e.g. surname, date of birth) where possible. For example, primary care records will be matched by extracting SystmOne records meeting the deterministic requirement that each of four fields (NHS number, surname, date of birth, gender) match a cohort participant record. Where unique identifiers are not available, iterative deterministic matching on the basis of multiple sets of non-unique identifiers will be used.

**6.2 Data storage and access management**

The central RDBMS hosted by BTHFT will store data obtained from all sources listed in Table 4.1. See Figure 4.1. Data from each source will be linked at the BSB person level and will be structured and maintained by BSB data managers as a long term strategic store to service cohort data capture, analysis and other research activities as necessary. The entire database schema and data will be backed up nightly.

- - 1. **Access to patient identifiable data within Bradford Teaching Hospitals NHS Foundation Trust**

Access to the RDBMS will be security controlled on the basis of BTHFT staff user roles managed by the BTHFT IT department. Access to patient identifiable data will only be permitted on a “need to know” basis, the test being whether the user needs to know the patient‟s identity in order to perform their role. For example, BSB data managers will have a “need to know” in order to clean and link participant data at the BSB person level. From time to time, BSB research assistants may need to contact BSB participants, in which case they have a “need to know” and will therefore have access to patient identifiable data.

Additional user roles will be created for users who need to access process and outcome data but who do not need access to patient identifiable data. Data extracts for such user roles will be anonymised. See Figure 4.1.

- - 1. **Access to patient identifiable data external to Bradford Teaching Hospitals NHS Foundation Trust**

There will be no access to patient identifiable data for individuals external to BTHFT without an additional Information Sharing Agreement signed by senior BTHFT staff and senior staff from the receiving organisation. Data extracts for BSB partner organisations and other external research groups carrying out approved secondary analyses will be anonymised. See Figure 4.1.

33

Born in Bradford‟s Better Start Cohort PROTOCOL V1 25.09.15

- - 1. **Anonymisation process**

Where required by BTHFT user role (see Figure 4.1) an anonymisation process will be applied to data extracts as follows:


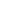


Personal identifiers (name, NHS number, hospital number, UPN) will be removed and replaced with pseudonymous identifiers (BSB Person ID).


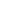


Potentially identifiable data fields (date of birth, address) will be removed, or if required for analysis, will be degraded to protect anonymity. For example, dates of birth can be converted to age, single residential addresses can be projected onto larger geographical units such as a Lower Layer Super Output Area (LSOA) or postcode sector.


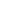


Rare categorical features (N<5) will be nullified or aggregated. For example, the categorical value for an ethnic group with fewer than 5 cohort members could be set to NULL. If there were fewer than 5 individuals with an annual income over £100,000, the income variable could be aggregated to a £50,000+ category to increase the number of members of that category.

**Figure 6.1 Cohort data store and access management at BTHFT**

|  | Staging tables |  |
| --- | --- | --- |
| Data Capture | clean, transform, |  |
| (see Table |  |  |
|  | link |  |

| Statistical analysis |  | BTHFT USER: |
| --- | --- | --- |
|  |  | statistician |
|  |  |  |
|  |  |  |


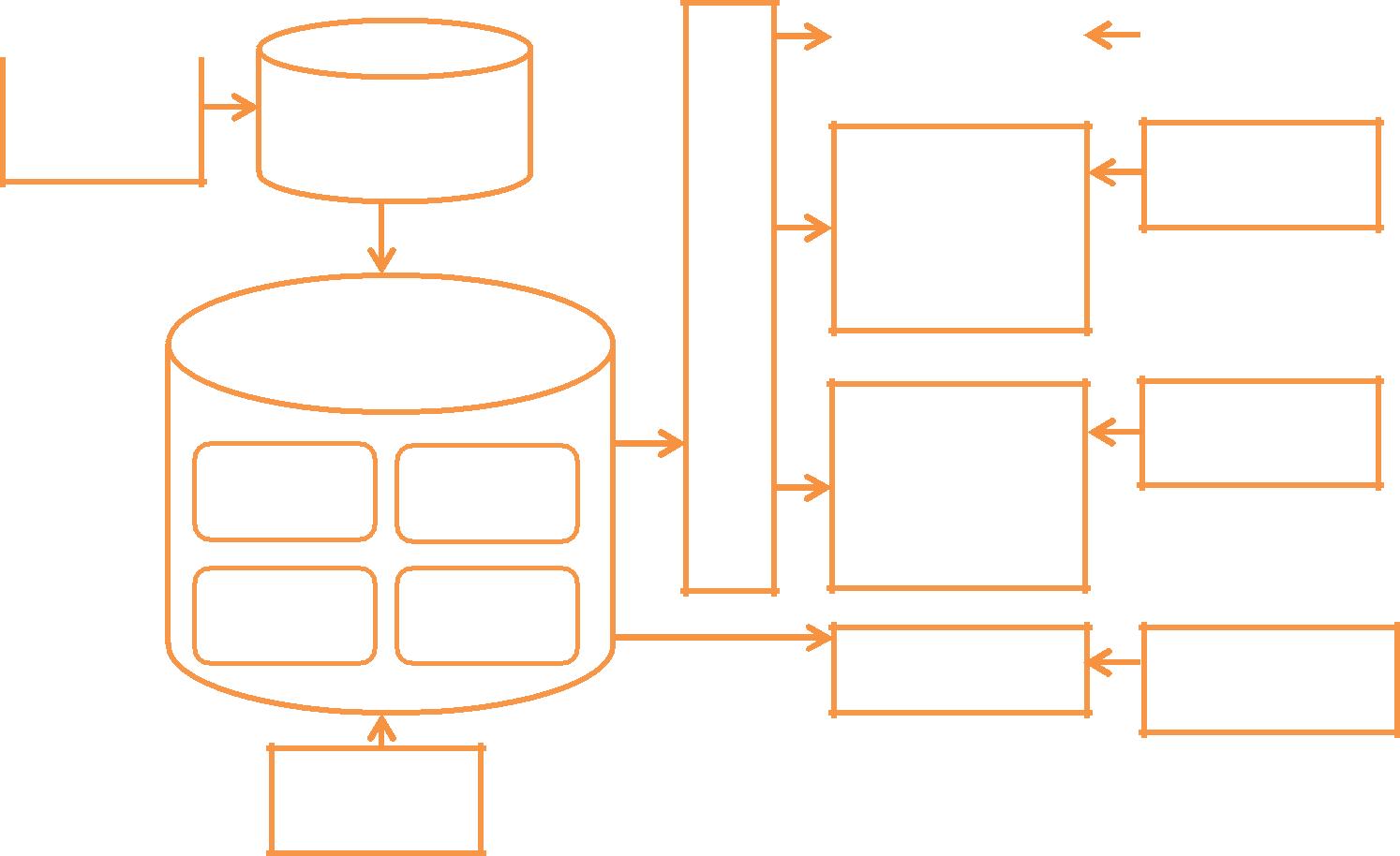


4.1)

Cohort RDBMS

| Primary | Data |
| --- | --- |
| Data | Linkage |
| Interventio | Biosample |
| n Data | Data |

| *Anonymisation process* |
| --- |

Reports: process tracking, funding application, study documentation

Data extract for BSB partner, or external secondary research proposal

BTHFT USER: project manager

BTHFT USER: data administrator

| Follow-up contact | BTHFT USER: |
| --- | --- |

research assistant

BTHFT USER: data manager

**6.3 Consent management**

Individual written consent (on a tablet or on paper) will be obtained from each adult cohort participant, for themselves and on behalf of the children from the current pregnancy. Capture of participant details and signature will be by tablet-based electronic form, with automated upload to the cohort RDBMS. Submission of the appropriate consent record to the cohort RDBMS will be required before a new participant record is created. Cohort participants may withdraw themselves and/or their children at any time by contacting the BSB Project Office by telephone or in writing. The participant withdrawal notification will be logged in the cohort RDBMS by BTHFT staff via web-based forms, along with reason for withdrawal and a record of whether the participant has also requested biosample destruction. The

34

biosample destruction process will be tracked in the cohort RDBMS, including the logging of written confirmation of biosample destruction by the holding laboratory. Following withdrawal, a participant will no longer be included in data requests for routine data linkage or intervention contacts or outcomes.

**Appendix 7: Project Management**

The BiBBS birth cohort is led by Prof John Wright and is coordinated by the BSBIH core team based within BiB, BTHFT, including the programme manager, principal research fellow, database manager, data analyst, project coordinator and researchers. The project management structure and membership of groups is outlined in Appendix 8.1.

- 1. **Project management groups**

**7.1.1 Cohort Theme Group**

This group includes the theme lead Prof J Wright, the Innovation Hub director, programme manager and principal research fellow, BiB programme manager, BiB Information analyst; and will meet at least monthly and work closely together to ensure the implementation of the BiBBS cohort. The group will have three areas of responsibility as follows:


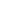


Effective set-up and recruitment into the cohort

Provide supervision and leadership for all members of the BiBBS cohort team

Each member will contribute to the delivery of the logistical aspects of the project which will include:

- Embedding the programme in routine NHS systems
- Development of supporting I.T. requirements
- Training & awareness of staff in their delegated area of responsibility Sample analysis and storage

The group will also:


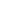


- Advise on and develop research methodology, including the biological sample bank
- Co-ordinate on research proposals for funding to grant giving bodies and collaborate as appropriate
- Evaluate the requirements for ethical clearance for the study

The group will also prepare applications for ethics approval, contribute to development of proposals for funding to grant giving bodies, and contribute to reports for BSB and the Big Lottery Fund.

There will also be Theme Management Groups, made up of the lead and steering group members of each theme. These groups will review proposals for publication relevant to each theme (Section 6).

- - 1. **Better Start Bradford Innovation Hub Programme Management Group**

This group will provide overall supervision of the Innovation Hub and all themes within that, including the cohort. Membership of the group includes the Innovation Hub director (KP), theme leads (CBC, PS, TB, MB, NS, JW), Programme manager (JD), principal RF (PB), BSB Director and relevant staff, BiB Programme manager (RM) and relevant BiB staff. The group will meet regularly. The objectives of the group are to:


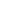


- To provide overall supervision of the Innovation Hub and to provide advice and guidance on the implementation of each of the Innovation Hub themes.


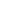


- To monitor and supervise the progress of the themes towards the Innovation Hub‟s interim and overall objectives, within the set time-frame.


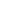


- To review at regular intervals relevant information from other sources (e.g. other related programmes/studies), and recommend appropriate action (e.g. changes to study protocols, stopping or extending the programme or individual studies within the programme).


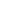


- To consider the implications of any recommendations made by the theme leads and BSB.
- To recommend appropriate action in the light of points 1, 2, 3 and 4 to ensure that the rights, safety and well-being of the participants are the most important considerations, and prevail over the interests of science and society.
- In light of 1, 2, 3 and 4 ensure that BSB are informed on the progress of the programme and that the PMG reports annually to the BSB Partnership Board.


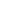


- To advise BSB on publicity and presentation of all aspects of the programme.


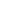


- To monitor and be responsible for data ownership and data release as detailed in the publication policy.
  - 1. **BiB Executive group**

The BiB executive group is already established and will take on additional responsibility for the BSBIH including:


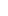


- To take overall responsibility for the implementation of the BiBBS cohort Oversee the strategic development of the cohort
- Review and provide scientific approval for collaborative studies Ensure compliance with good research governance
- Provide supervision and leadership for the BiBBS cohort team Co-ordinate the different groups involved in undertaking the programme To provide financial reports to the Trust Board
- To act as custodians of the biobank samples

This group will provide the first point of contact for researchers seeking approval to collaborate or gain access to the data. Potential nested study applications will be reviewed by the Executive Group for consideration using a proforma.

- - 1. **BSB Partnership Board**

The purpose of the BSB Partnership Board is to govern the strategy, policies and delivery of the BSB Programme. The Innovation Hub is required to report to the BSB partnership board at least annually on progress against agreed milestones. The BSB Partnership terms of reference are provided in Appendix 8.2.

- - 1. **Community Representatives Advisory Group**

This group includes representatives from the community in BSB areas, including parents and grandparents, councillors, representatives from community organisations. Members of the research team will meet the community representatives regularly to obtain feedback on research plans and data collection tools, including the participant information sheet and the baseline questionnaire, and to discuss ideas for the research.

- - 1. **Independent scientific steering committee**

This group will provide independent scientific oversight to the programme. Specific roles include: Monitoring progress of the research

Providing strategic scientific direction

Acting as an appeal panel where necessary

Promoting transparency in project management

Adjudicating on requests for access to data

This committee will act as an independent body and maintain a broad remit in terms of reviewing new questions; new measures including biological samples; new studies and nested studies; protocol based prospective measures that may need to be fed back and participant information. It will consider key ethical matters and ensure relevant policies are in place and adhered to, to ensure safeguarding of participants. The group will report directly to the BiB Executive Group.
